# A high-throughput integrated microfluidics method enables tyrosine autophosphorylation discovery

**DOI:** 10.1038/s42003-019-0286-9

**Published:** 2019-01-30

**Authors:** Hadas Nevenzal, Meirav Noach-Hirsh, Or Skornik-Bustan, Lev Brio, Efrat Barbiro-Michaely, Yair Glick, Dorit Avrahami, Roxane Lahmi, Amit Tzur, Doron Gerber

**Affiliations:** The Mina and Everard Goodman Faculty of Life Sciences and the Institute of Nanotechnology and Advanced Materials, Bar-Ilan University, Building #206, Ramat-Gan, 5290002 Israel

## Abstract

Autophosphorylation of receptor and non-receptor tyrosine kinases is a common molecular switch with broad implications for pathogeneses and therapy of cancer and other human diseases. Technologies for large-scale discovery and analysis of autophosphorylation are limited by the inherent difficulty to distinguish between phosphorylation and autophosphorylation in vivo and by the complexity associated with functional assays of receptors kinases in vitro. Here, we report a method for the direct detection and analysis of tyrosine autophosphorylation using integrated microfluidics and freshly synthesized protein arrays. We demonstrate the efficacy of our platform in detecting autophosphorylation activity of soluble and transmembrane tyrosine kinases, and the dependency of in vitro autophosphorylation assays on membranes. Our method, Integrated Microfluidics for Autophosphorylation Discovery (IMAD), is high-throughput, requires low reaction volumes and can be applied in basic and translational research settings. To our knowledge, it is the first demonstration of posttranslational modification analysis of membrane protein arrays.

## Abstract (web summary)

Hadas Nevenzal, Meirav Noach-Hirsh et al. present a method for direct detection and analysis of tyrosine autophosphorylation using integrated microfluidics and arrays. They show that their method can be applied to soluble and transmembrane tyrosine kinases as well as to membranes in vitro.

## Introduction

Protein arrays complement mass-spectrometry in proteomic research. Much like DNA microarrays, standard protein arrays are essentially a matrix spotted with thousands of proteins^1,2^. Each protein is equally represented and virtually the only one in its spot, thereby circumventing the main challenge in mass-spectrometry-based analyses, i.e., protein/peptide relative abundance. This challenge is much heightened in the context of protein posttranslational modification (PTM) discovery. First, PTMs are reversible, highly dynamic, and often occupying only a small fraction of the target protein. Second, PTMs are identified on their unique target peptides, which can be low abundant by themselves. Standard protein arrays, however, rely on pre-purified recombinant proteins and thus, incompatible with insoluble and other biochemically challenging proteins. Moreover, the spotted proteins are aged in non-physiological conditions for weeks if not months before use, raising concerns about protein folding and functionality.

Integrated microfluidics paved the way to freshly expressed protein arrays^3,4^. The microfluidic platform enables expression of thousands of proteins in reticulocyte lysates. A set of pneumatic valves, allow compartmentalization of each target protein in individual unit cells, overriding major limitations and caveats of open protein arrays^1,2,5^. This technology was originally developed for screening direct protein–protein interactions^3,4^. Interaction between proteins and nucleic acids was also showed^6^^,7^. More recently, the platform was proven to be compatible also with protein PTM analyses^8^. In that study, we applied recombinant enzymes or active cell extracts to the chip to promote PTM of fresh proteins in quasi-cellular environments. Both the target protein and the protein modifier were then quantified colorimetrically to derive a normalized PTM signal. Tyrosine (Tyr) phosphorylation, ubiquitination, and ubiquitin chain preference was demonstrated^8^. Although functional as substrates for protein interactions and PTMs, it is still unclear whether the arrayed proteins maintain intrinsic catalytic activity. This is not a marginal distinction because enzymes are expected to be considerably more demanding in terms of folding and functionality. Arrays of functional enzymes for high-throughput activity assays are valuable for basic and translational research; in fact, targeting enzymes is a major strategy in drug design^9–11^.

Autophosphorylation is a biochemical process in which a phosphate (P) group is added to a protein kinase by itself^12^. This molecular node is a ubiquitous mediator between extracellular cues and signal transduction pathways associated with a great variety of normal and pathological processes ranging from cancer to complex developmental disorders. We evaluated the potency of integrated microfluidic as an enzymatic array, focusing on autophosphorylation of soluble and membrane Tyr kinases.

## Results

### On-chip autophosphorylation of soluble p-Tyr

**Fig. 1:**
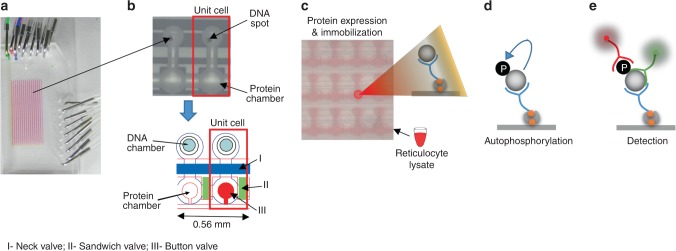
Device and strategy. Integrated microfluidic device combined with His/Myc-double-tagged ORF library spotted on glass (the observed DNA in the picture encodes for FRK protein), allow parallel expressions of thousands of proteins ready for biochemical assays. Each unit cell comprises DNA and protein chambers isolated by valves. **a** The entire microfluidics device. **b** Target proteins are expressed in DNA chambers following incubation with reticulocyte lysate, diffused, and immobilized in protein chambers via His tag. Overall, 10 μl reagents are sufficient to cover a chip of thousands unit cells. **c** Proteins are expressed in mammalian cell lysates. Thus, enzymes with inherent autophosphorylation activity are expected to be functional and undergo autophosphorylation during expression. **d**, **e** Arrayed proteins and phosphorylated Tyr (P-Tyr) are both quantified in situ using Cy3-coupled anti-Myc antibodies and Cy5-coupled anti-phosphorylated-Tyr antibodies, respectively. Finally, a net autophosphorylation signal is determined

The shift from Tyr phosphorylation to Tyr autophosphorylation assay on our microfluidic platform^8^ is conceptually simple (Fig. 1). In brief, a device combining a microarray spotted with a double-tagged cDNA library of interest, and bilayer microfluidics that are based on polydimethylsiloxane (PDMS) lithography, is assembled. This design generates a set of channels regulated by pneumatic valves capturing each of the spotted cDNA molecules in an individual chamber, i.e., DNA chamber (Fig. 1b). Reticulocyte lysate is then applied to all DNA chambers for in vitro transcription and translation. The resulted in vitro transcription and translation product in each unit cell diffuses to the protein chamber and is immobilized via affinity tag (Fig. 1c). In vitro transcription and translation products with intrinsic phosphorylation activity potentially undergo autophosphorylation during the expression process (Fig. 1d). Following washing, each unit cell is enriched with a single kind of protein whose level and P-Tyr level are both quantified in situ by immunofluorescence to determine total or net autophosphorylation on chip (Fig. 1e). We hypothesized that this course of events has the potential to specifically detect autophosphorylation if the arrayed proteins are catalytically active.

**Fig. 2:**
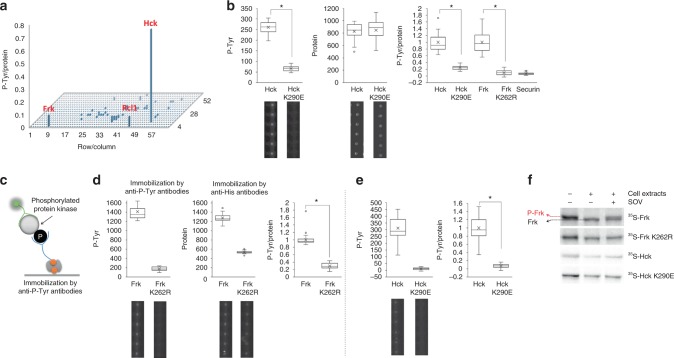
Large-scale Tyr autophosphorylation analysis of freshly expressed protein arrays. **a** An array of 882 human proteins was expressed on chip in quadruplicates. Protein and P-Tyr signals were quantified as described in Fig. 1. Each bar represents an average P-Tyr-to-Protein ratio of a single protein (*n* = 4). Top 3 hits are indicated. Source data is provided in Supplementary Figure 2. **b** Assay validation. Hck and Frk kinases, their inactive variants (K290E; K262R) and the non-kinase protein Securin, were expressed in tube, deposited on chip, and assayed for autophosphorylation activity as described in Fig. 1. Average P-Tyr normalized to protein levels are shown (*n* = 17–36; *P < 0.05). Data are normalized to maximal activity for each kinase. Absolute P-Tyr and protein levels, and representative raw data are shown for wild type and inactive Hck to clarify the methodology. Source data is provided in Supplementary Figures 4 and 5. **c** Schematic presentation of anti-P-Tyr immunoprecipitation. **d**, **e** Immunoprecipitation of autophosphorylated proteins on chip. **d** Frk wt or **e** Hck wt and their kinase dead derivates (K262R, K290E respectively) were immobilized on chip using biotinylated anti-P-Tyr antibody. Proteins expression value was evaluated using anti-His antibody for immobilization and anti C-myc. Representative raw data is shown below (*n* = 34 for (**d**) and *n* = 63 (**e**); *P < 0.01). Results were normalized to each protein expression level (P-Tyr/Protein) as well as to maximum activity (Frk or Hck phosphorylation level). Source data is provided in Supplementary Figures 6 and 7. **f** ^35^S-labeled Frk wt, Hck wt and their kinase dead derivates were in vitro transcribed and expressed using rabbit reticulocyte lysate and incubated for 30 min, 37 °C in parallel with HEK293 cell extracts supplemented with sodium orthovanadate (1 mM), as depicted in the plot. Protein’s mobility shift was assayed by SDS–PAGE and autoradiography. Source data is provided in Supplementary Figures 8 and 9

We base this hypothesis on the assumption that little or no Tyr kinase activity is present in the reticulocyte lysate. To test for specific autocatalytic activity, we first measured the intrinsic P-Tyr signals of 882 human proteins on chip, underrepresented for Tyr kinases (see Supplementary Table 1). We used FRK and HCK, known non-receptor Tyr kinases, as positive controls. After normalization to protein levels, mean P-Tyr-to-protein ratios were plotted (Fig. 2a; Supplementary Figure 1. Source data for Fig. 2a is shown in Supplementary Figure 2). Only the following three proteins: Hck, Frk, and Rcl1 (in that order), exhibited substantial P-Tyr signals. The first two proteins are positive controls with known autophosphorylation activity^13,14^, in line with our autophosphorylation hypothesis. Overall, this experiment demonstrated that the reticulocyte lysate has very little intrinsic phosphorylation activity as demonstrated by hundreds of proteins on the array with no P-Tyr signal. Only a single protein, Rcl1, out of 800 demonstrated false positive activity. Further analysis of this protein, a non-kinase RNA 3′-terminal phosphate cyclase-like protein^15^, indicated that the false positive activity stemmed from DNA contamination (Supplementary Figure 3A). We isolated Rcl1 DNA and expressed it again. As expected Rcl1 had no kinase activity (Supplementary Figure 3B). The DNA contamination was introduced in the assembly PCR reaction.

The bright P-Tyr signal observed for both Hck and Frk was interpreted as autophosphorylation. To confirm that, Hck and Frk carrying a defective ATP-binding sites were generated. These mutants are considered catalytically inactive; yet, they can still be phosphorylated by other kinases that might be present in the reticulocyte lysate. In vitro transcription and translation products of Hck and Frk, their inactive derivate, and the non-kinase protein Securin^16^, were assayed for autophosphorylation. The latter was used as a negative control with which we could determine background P-Tyr levels. As shown in Fig. 2b, P-Tyr signals of the two mutant kinases dropped by 75 to 90%, nearly reaching a background signal. Noteworthy, a faint, albeit noticeable, P-Tyr signal observed for inactive Hck. Owing to the overall low Tyr kinase activity in reticulocyte lysates, as demonstrated for hundreds of proteins (Fig. 2a), it is easy to speculate that Hck carrying a Lys to Glu mutation at position 290 can still maintain a weak catalytic activity. Regardless, the majority of Hck’s P-Tyr signal resulted from autophosphorylation per se. We independently validated the autophosphorylation of Frk and Hck by immunoprecipitation of these two proteins with anti-p-Tyr antibody (Fig. 2c–e). We further validated Frk’s and Hck’s autophosphorylation using a mobility shift assay by SDS–PAGE (Fig. 2f). Unfortunately, this assay was not informative for Hck. Together, the results presented in Fig. 2d–f validated the potential of our platform to specifically detect Tyr autophosphorylation.

### Sensitivity and specificity of the microfluidic platform

**Fig. 3:**
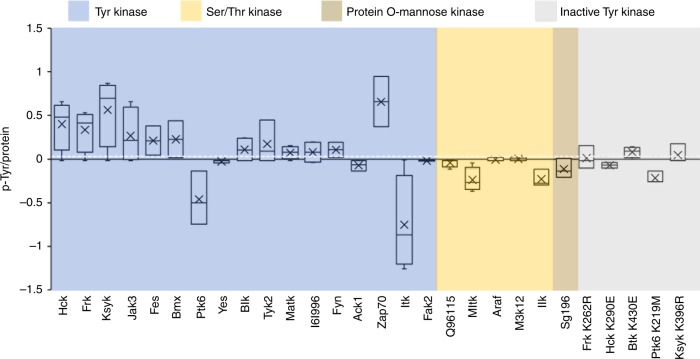
Autophosphorylation analysis of Tyr kinase array. An ORF library comprising 17 soluble Tyr kinases and 11 negative controls (5 inactive Tyr kinases and 6 non-Tyr kinases) was generated by assembly PCR. ORFs were spotted in quadruplicates. A freshly expressed protein array was generated and assayed for autophosphorylation as described in Fig. 1. Bars represent an average and standard deviation values of P-Tyr signals (*n* = 4) after background P-Tyr signal subtraction and normalization to protein level. A cutoff value of 0.05 was calculated by receiver operating characteristic analysis

In order to determine the sensitivity and specificity of the assay numerically, we arrayed 17 non-receptor Tyr kinases with known autophosphorylation activity alongside 11 negative control including Ser/Thr kinases, protein-*O*-mannose kinase, and inactive Tyr kinases. Mean P-Tyr levels normalized to protein levels were plotted and a cutoff value for autophosphorylation activity was calculated using receiver operating characteristic analysis^17^. Normalized P-Tyr levels of 12 out of 17 Tyr kinases were above cutoff value (Fig. 3). On the other hand, only 2 out of 11 negative controls exhibited P-Tyr signals above the threshold. Overall, sensitivity and specificity of the assay were calculated to be 0.7 and 0.8, respectively (Table 1). Taken together, results in Figs. 2 and 3 demonstrate an on chip assay for autophosphorylation of soluble Tyr kinases. These results also provide definite evidence that our freshly expressed protein arrays are catalytically active.

**Table 1:** Sensitivity and specificity of the mobility shift assay

True positive: 12	False positive: 2
False negative: 5	True negative: 9
Sensitivity: 0.7	Specificity: 0.8

### On chip autophosphorylation of membrane protein

**Fig. 4:**
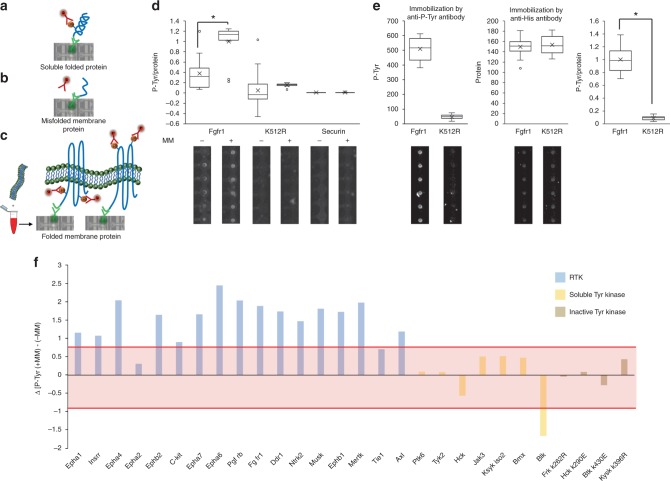
A functional array of receptor Tyr kinases (RTKs). Challenges and mitigation. Soluble protein kinases properly folded in cell lysates exhibiting specific autophosphorylation activity that can be detected by anti-P-Tyr antibodies (DNA spot in the picture encodes for FRK protein) (**a**). Conversely, membrane proteins are misfolded with limited and/or ectopic kinase activity, if at all (**b**). A proper folding of membrane proteins is achieved in reticulocyte lysate supplemented with microsomal membranes. Following immobilization and detection, bona fide autophosphorylation activity can be assayed on chip (**c**). The observed DNA spots in **b** and **c** encode for the expression of wild-type FGFR1. **d** Assay validation. Wild type and inactive mutant (K512R) Fgfr1, and Securin (negative control), were expressed in reticulocyte lysate supplemented with microsomal membranes (+MM) or mock (−MM), deposited on chip and assayed for autophosphorylation. Average P-Tyr to protein levels are shown (*n* = 11–25; *P < 0.01). Data are normalized to maximal activity. Representative raw data showing P-Tyr detection are shown. Source data is provided in Supplementary Figures 10–12. **e** Immunoprecipitation of autophosphorylated membrane protein on chip. Fgfr1 and kinase dead (K512R) were immobilized on chip using biotinylated anti-P-Tyr antibody. Proteins expression value was evaluated using anti-His antibody for immobilization and labeling with Green Lysine. Representative raw data is shown below (*n* = 20; *P < 0.01). Results were normalized to each protein expression level (P-Tyr/Protein) as well as to maximum activity (Fgfr1 phosphorylation level). Source data is provided in Supplementary Data Figure 12. **f** A global dependency of RTK activity assays on membranes revealed on chip. An ORF library comprising 17 RTK, 7 soluble Tyr kinases, and 4 inactive Tyr kinases was spotted on chip in quadruplicates. Proteins were expressed in reticulocyte lysate supplemented with microsomal membranes (MM) or mock (−MM). An average P-Tyr signal from three experiments was determined for the arrayed proteins, and the difference: [P-Tyr^(+MM)^] - [P-Tyr^(−MM)^] was plotted. A cutoff (±0.84) marking significant impact of microsomal membranes on autophosphorylation activity was determined by receiver operating characteristic analysis as 3× standard deviation from P-Tyr values calculated for inactive kinases. Membrane effect on autophosphorylation is considered significant if P-Tyr values are either above (positive cutoff) or below (negative cutoff) the red region

Membrane proteins mediate essential cellular processes, first and foremost cell signaling and communication. They are the key for host–pathogen interactions, and profoundly linked to human disease and disorders. Located at the cell surface, receptor Tyr kinases (RTKs) are also considered attractive targets for drug-based therapy^18,19^. This highlights the need for systematic approaches for studying RTKs and membrane proteins in general. The technological challenge, however, is high; arrays of pre-purified proteins are restricted to soluble proteins. Even if freshly expressed, most membrane proteins cannot fully fold in an aqueous solution and are dysfunctional (illustrated in Fig. 4a, b). This limitation can, in principle, be overcome by applying microsomal membranes to the protein translation solution (Fig. 4c)^20^. Relying on this technique, we have recently reported the first membrane protein array for protein interaction studies^21^. The catalytic activity of the arrayed proteins, however, remained unknown.

PTM analysis, let alone phosphorylation, has never been demonstrated on a membrane protein array of any kind. We decided to evaluate the capacity of our platform to detect autophosphorylation of RTKs. First, we analyzed fibroblast growth factor receptor 1 (Fgfr1), a well-known RTK with autophosphorylation activity^22^. A His/Myc tagged Fgfr1 was translated in lysates containing microsomal membranes or mock, immobilized on the surface of the protein chambers, and assayed for autophophorylation on chip. A bright P-Tyr signal was measured only for Fgfr1 containing microsomal membranes (+MM) (Fig. 4d). This signal was nearly 3-folds higher relative to membrane-free Fgfr1 (−MM), demonstrating a strong dependency of Fgfr1’s activity in vitro on membranes. Comparison with an inactive mutant of Fgfr1 (K512R) confirmed that the P-Tyr signal results almost exclusively from autophosphorylation and not phosphorylation by external factor or artefactual signal. We validated the autophosphorylation of Fgfr1 by immunoprecipitation with anti-p-Tyr antibody (Fig. 4e).

Next, a library of 17 RTKs, all of which known to undergo autophosphorylation, were expressed in quadruplicates on chip using reticulocyte lysate supplemented with microsomal membranes (+MM) or mock (−MM). Eleven soluble Tyr kinases, 4 of which are inactive mutants, were also arrayed as negative controls and to determine non-specific background signals. P-Tyr signals were quantified for the 28 proteins expressed in the two conditions (Supplementary Figure 13). Mean P-Tyr^(−MM)^ levels were subtracted from the matching P-Tyr^(+MM)^ signals, and the ΔP-Tyr^([+MM]-[-MM])^ values were plotted (Fig. 4f). Proteins within upper and lower cutoff lines (red zone) presented no significant changes in autophosphorylation levels whether or not membranes were present. Thresholds were determined by receiver operating characteristic analysis. B lymphocyte kinase (Blk) was the only soluble kinase for which we noticed a significant impact of microsomal membranes on autophosphorylation. Interestingly, this effect was negative; P-Tyr signal of Blk1 was reduced in the presence of membranes (Fig. 4f), suggesting that membranes might have an inhibitory impact on Blk catalytic activity in our assay. More importantly, microsomal membranes increased autophosphorylation activity in 15 out of 17 arrayed RTKs (88%), affirming the indispensability of this reagent for RTK activity assays in vitro. Altogether, the data presented in Fig. 4 demonstrate the power of our platform in studying Tyr autophosphorylation of RTKs on chip in a physiological-relevant context, as well as validate the functionality of our membrane protein array by showing a specific, well-characterized, enzymatic activity.

### Autophosphorylation of Ror2 a controversial pseoudokinase

**Fig. 5:**
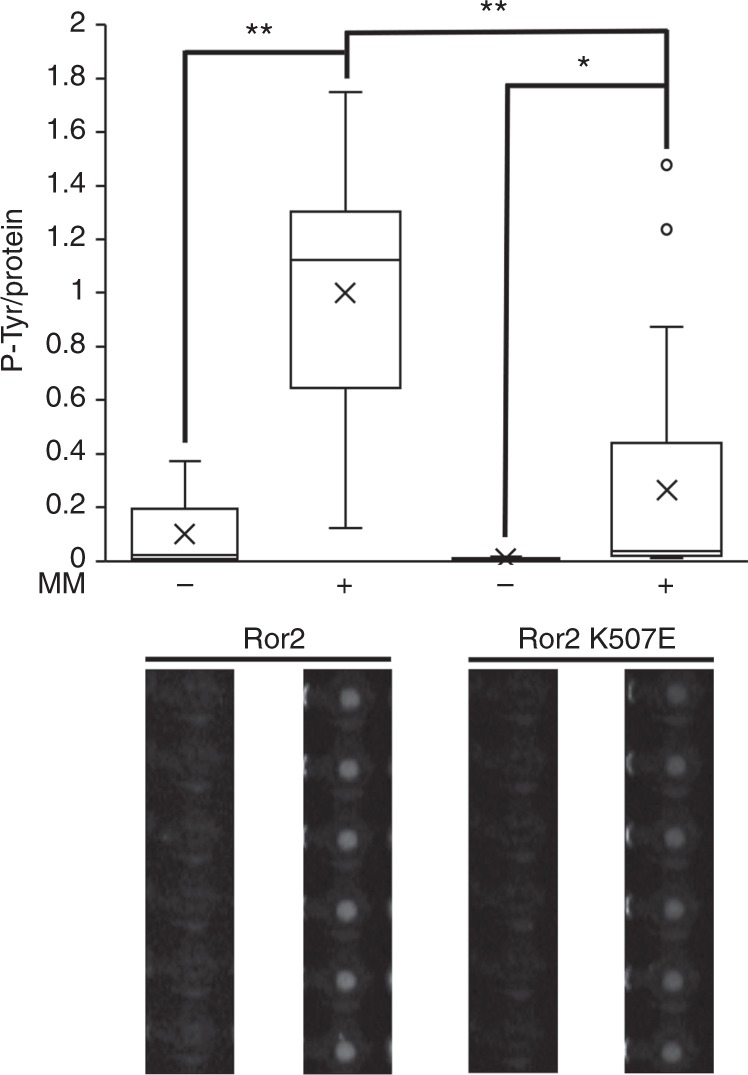
A membrane-dependent autophosphorylation activity for Ror2. Myc/His-tagged Ror2 and its inactive derivate (Ror2 K507E) were expressed in reticulocyte lysate supplemented with microsomal membranes (MM) or mock (−MM), and immobilized to protein chambers for an on-chip autophosphorylation activity assay. An average P-Tyr/Protein level ratio is shown (*n* = 13; *P < 0.05, **P < 0.001). Data are normalized to maximal activity. Representative raw data are shown. Error bars = 1 SE. Source data is provided in Supplementary Figure 14

Receptor Tyr kinase-like orphan receptor (Ror) subfamily of RTKs includes two related proteins, Ror1 and Ror2, both functioning in the Wnt signaling pathway^23,24^. Mutations in Ror2 associate with human disease including Robinow and Brachydactyly type B skeletal syndroms^25,26^. Overexpression of the protein has been linked to cancer development and prognosis^27,28^. Ror2 intrinsic activity is controversial^29,30^. In vitro studies combined with phylogenetic analyses showed that Ror2, different from its *C*. *elegans* homologue CAM-1, nearly lost its innate catalytic activity, classifying it as a catalytically deficient RTK-like pseodokinase^31,32^. This notion, however, is challenged by contradicting evidence^33,34^. Intrigued by this debate, we utilized our platform to investigate the potential of Ror2 to undergo autophosphorylation in vitro. To this end, we tested Ror2 autophosphorylation on chip in the presence of microsomal membranes or mock. As shown in Fig. 5, a profound P-Tyr signal was observed for Ror2 in a membranous environment. This signal dropped by nearly 75% in Ror2 mutant lacking ATP-binding site (Ror2 K507E), thus, confirming exclusive detection of autophosphorylation. The dependency of Ror2 activity on membranes was remarkable; in fact, the impact of membranes was more dramatic than Lys to Glu substitution at the of ATP-binding site. These results indicate for an intrinsic kinase activity for Ror2 as well as the indispensability of membranes in RTK activity assays in vitro.

## Discussion

In this study, we developed and utilized an integrated microfluidics platform for Tyr autophosphorylation discovery (IMAD). The method is large scale, quantitative, compatible with both soluble and membrane kinases, and benefits from the inherent simplicity and versatility of in vitro assays while still maintaining physiologically relevant context. The sensitivity and specificity of the assay falls well within the acceptable standard of advanced large-scale analyses (see, for example, ref. ^35^), and can also be appreciated by the standard phosphatase inhibitor response (Supplementary Figure 15). We are not aware of equivalent proteomic assay.

About half of human Tyr kinases are RTKs; most, if not all, involved in pathological signaling cascades^36^. Surrounded by membranous environment, RTKs were proven to be active in our apparatus. To our knowledge, this is the first systematic assay for RTK autophosphorylation as well as the first PTM analysis of membrane protein arrays. The data demonstrate a strong dependency of RTK activity (but not expression) on membranous environment in vitro. It also emphasizes the caveats of RTK activity assays taking place in aqueous solutions. At this juncture, it is important to note that methodological differences can clearly account for the conflicting arguments regarding RTK activity, with Ror2 being no different. In our hands, Ror2 autophosphorylation was unambiguous. Furthermore, we note that Ror2 overexpression has been linked to cancer development^37,38^. Bridging the gap between in vitro and in vivo, the platform presented here is useful in screening compounds that block autophosphorylation of Ror2 and other Tyr kinases for cancer therapeutics.

Tyr kinases, in particular RTKs, are normally activated by ligand/antibody-mediated dimerization^12^. For the most part, these molecules are unlikely to be available in reticulocyte lysates. And yet, Fgfr1, Ror2, and nearly all other RTKs tested here were catalytically active. We believe that the high local concentration of the kinases immobilized on the protein chamber surface, let alone in microsomes, circumvent ligand-induced dimerization, and effectively support meaningful autophosphorylation. This feature is advantageous as it greatly simplifies the assay. That being said, autophosphorylation may not reach its maximum potential without specific ligand activation. In accord with this notion, we show that autophosphorylation of the proto-oncogene c-Kit, although noticeable, is improved by adding a ligand (Supplementary Figure 16). The potential of our platform in studying ligand–receptor relationships is clear; nevertheless, outside the scope of this current research.

Autophosphorylation is divided to trans where each kinase molecule is phosphorylated by the dimerized partner, and *cis* where the kinase molecule is targeted by its own catalytic domain^12^. Distinguishing between these two forms is important for understanding the mechanism by which the kinase is activated. This is a challenging task by all means and beyond the capacity of our current apparatus. On the other hand, our platform is optimal for distinguishing Tyr phosphorylation from autophosphorylation. Relying on spotted cDNA molecules, arraying a library of inactive kinases is straightforward. Because kinase dead mutants can still be targeted by other active kinases, P-Tyr signal in this case represents phosphorylation. An example highlighting this concept is shown for Btk (Supplementary Figure 17).

Detection of autophosphorylation on chip is based on immunolabeling. The method is currently limited to Tyr because of the poor specificity of global anti-phospho Ser/Thr antibodies. In view of mammalian Ser/Thr kinases being ~80% of the kinome, the motivation to mitigate this limitation in the future is high. Further we note that autophosphorylation, although widespread, is not the only known automodification; autoribosylation, autoadenylylation, autoubiquitination, and autoneddylation have been reported in various processes, some related to human disease^39–42^. Our protein arrays are functional. As long as the modifier molecule is detectable in situ and the arrayed protein is catalytically active by itself, the platform used here can be adjusted for discovery of other auto-PTMs. Overall, we believe that the methodology reported here holds a great potential for basic and translational research.

## Methods

### Microfluidics device fabrication

Integrated two-layer microfluidic devices were designed in AutoCAD2013 (Autodesk, Inc., Mill Valley, CA). Mold fabrication was performed using soft lithography and chrome mask, as previously detailed^8^. These molds were used for fabricating microfluidic device by casting silicone elastomer polydimethylsiloxane (PDMS; SYLGARD 184®, Dow Corning). Each microfluidics device consists of two aligned PDMS layers, the flow and the control layers.

### Surface chemistry

Biotinylated-BSA (1 μg/μl, Thermo) was flowed for 30 min through the device, binding the BSA to the epoxy surface. On top of the biotinylated-BSA, 0.5 μg/μl of Neutravidin (Pierce, Rockford, IL) was added for 30 min. The button valve was then closed, and biotinylated-PEG (1 μg/μl, Nanocs) was flowed over for 30 min, thus passivating the rest of the flow layer. Following passivation, the button valve was released and a flow of 0.2 μg/μl penta-His biotinylated (Qiagen, Venlo, Netherlands) or 0.01 µg/µl anti-Myc biotinylated antibodies (Cell Signaling, Danvers, MA, USA) were applied. The antibodies bound to the exposed Neutravidin, specifically to the area under the button, creating an array of anti-His- or anti-Myc tag. PBS buffer was used for washing between each surface chemistry step.

### Generating expression library

A library of Tyr kinase and controls open reading frames (ORFs) was generated. Most of the genes were cherry picked from the Open BiosyStem’s library of human ORFome, while others were purchased from Addgene (Cambridge USA). The ORF’s library used to create synthetic linear genes by two steps PCR. The ORFs were used as a template. ORF’s were then double tagged using a 5′ primer with-Myc tag, and a 3′ primer with a His tag. The second PCR step was performed with extension primers containing T7 promoter (5′) and T7 terminator (3′) and the PCR product from step 1 as a template. The PCR products were filtered with Wizard SV Gel and PCR Clean-Up System (Promega, Madison, USA) and eluted with 40 µl DDW. All PCR reactions were performed with high-fidelity hot start DNA polymerase KAPA (KAPA Biosystems, Wilmington, USA). DNA point mutations, for creating inactive kinases, were produced using QuikChange Lightning kit (Agilent, Santa Clara, USA). The following point mutations were made to generate inactive kinases: Hck K290E; Btk K430E; Ror2 K507E; Frk K262R; Ptk6 K219M; Ksyk K397R^43^; Fgfr1 K512R. Mutation sites are for the most part at the ATP-binding site, as noted in the UniProt database. Primer sequences are presented in Table S2. All open reading frames and mutations used in this paper were validated by DNA sequencing. For oligos used in mutagenesis and assembly PCR see Supplementary Table 2 and 3.

### DNA arraying and device alignment

Synthetic linear DNA samples were mixed with 1.25% D-trehalosedihydrate and 0.125% of polyethylene glycole (Sigma, Israel) in 384-well plates (Greiner bio-one) and spotted in quadruplicates on epoxy coated glass slides (CEL Associates) using a MicroGrid 610 microarrayer (Bio Robotics) equipped with SMT-S75 silicone pins (Parallel Synthesis). Next, the DNA array was aligned to PDMS using a µDAS semiautomatic aligner^44^.

### Protein expression and immobilization

For on-chip expression, a pre-mixed reticulocyte lysate supporting protein expression by T7 promoter (12.5 μl) with or without microsomal membranes was flowed into the DNA chambers, following surface chemistry. See more details in refs. ^8,21^. Alternatively, in vitro translation took place in tube, and the lysate was flowed directly into the protein chambers for immobilization. Detection of the immobilized proteins was based on immunofluorescence with either Cy3-coupled anti-Myc antibodies (1/100 dilution; Sigma Israel) or Alexa-Fluor 647-coupled anti-His antibodies (Qiagen, Venlo, Netherlands). The detection antibodies were flowed into the device, and incubated with the immobilized proteins under the button for 30 min at RT, followed by a wash with PBS buffer. Protein expression levels were determined with a microarray scanner (LS Reloaded, Tecan, Männedorf, Switzarland) using a 532 nm laser and 575/50 nm filter for Cy3-anti-Myc, and 633 nm laser and 692/40 filter for Alexa 647-anti-His antibodies.

### On-chip Tyr kinase assay

After protein expression and immobilization under the buttons in the protein chambers, the button valve was open, and the expressed kinases were further incubated for 30 min at 37 °C. After wash, the kinases were incubated for 30 min at RT with anti-P-Tyr antibodies coupled to Alexa-Fluor 647 or alternatively to Alexa-Fluor 488 (Cell signaling, Danvers, MA, USA). Next, the button was closed and the chip was washed to remove unbound antibodies. Autophosphorylation levels were determined with the microarray scanner using a 633 nm laser (emission filter: 692/40) or 488 nm laser (emission filter: 535/25).

The phosphorylation level of soluble kinases was normalized to the protein expression level giving P-Tyr-to-protein ratio, and to the background P-Tyr signal, i.e., the average P-Tyr/protein value measured for negative controls. P-Tyr levels of membrane kinase arrays was normalized to background P-Tyr signal. For all experiments, the signal resulting from anti-P-Tyr non-specific binding was determined on-chip. These background values were then subtracted from the gross phosphorylation signal. Cutoff values were determinate using receiver operating characteristic analysis or standard deviation.

### Immunoprecipitation of autophosphorylated proteins on chip

Kinases were expressed by in vitro transcription and translation kit (Promega, Madison, USA) and labeled with FluroTect Green Lysine (Promega, Madison, USA). The kinases were flowed into the device and immobilized on the surface under the button within the protein chamber with either anti-His (Qiagen, Venlo, Netherlands) or phospho-biotin antibody (Cell Signaling, Danvers, MA, USA). After washing, autophosphorylation or expression levels were determined with the microarray scanner using a 488 nm laser (emission filter: 535/25).

### Mobility shift assay

Kinases were expressed by in vitro transcription and translation kit (Promega, Madison, USA) and labeled with Methionin 35S (Perkin Elmer, Israel). The kinases and their derivate mutants were incubated with HEK293 cell extract, supplemented with mock or 1 mM Sodium Orthovanadate for 30 min at 37 °C. Samples were resolved by SDS–PAGE and visualized by autoradiography using Fuji phosphorimager BAS-2500.

### Image and data analysis

The signal of Tyr phosphorylation and kinase expression were measured under the button. Each experiment was performed on at least two separate devices, with four technical repetitions for each protein in each device. LS Reloaded microarray scanner (Tecan) and GenePix7.0 (Molecular Devices) image analysis software were used for all experiments analysis. We consider the signal measured around the button valve as the background since no protein immobilization is expected there. However, some background is always observed. The background signal in our microfluidic protein arrays results from non-specific ligation of antibodies to the surface. For each wavelength scanned, we subtracted the corresponding background signal around the buttons in a ring the size of 2R with 2-pixel spacing (see supplementary material in Noach-Hirsh et al.^8^). MCP. Protein expression levels and autophosphorylation were detected using fluorescent antibodies: 532 and 635 nm emission, respectively. Scanning in the two wavelengths was performed in a serial manner. No crossing signals were observed between the two wavelengths in control experiments. The uniformity of the immobilized protein spots was analyzed and also manually curated. The level of median signal, for each protein, was calculated using four points, which were then averaged. Using median signals reduced noise from spot uniformity. These signals served to determine protein expression levels. As the phosphorylation level of each kinase was normalized to its protein expression level, the issue of global uniformity over the entire array was negligible. We commonly observed variability of up to 20% between spots for thousands of proteins and hundreds of protein arrays. Each time the variability was higher, manual curation demonstrated a spot that was determined invalid (e.g. the DNA was spotted outside the DNA chamber and no protein was expressed in one of the four repeats).

### Cell culture

HEK293 cells were maintained in tissue culture plates containing Dulbecco’s Modified Eagles Medium (DMEM) supplemented with 10% fetal bovine serum (FBS), 2 mm l-Glutamine, and penicillin (100 u)/streptomycin (0.1 mg/ml) (all reagents were purchased from Biological Industries, Kibbutz Beit Haeemek, Israel). Cells were maintained at 37 °C in a humidified 5% CO_2_ environment.

### Active cell extract preparation

HEK293 cells were lysed in swelling buffer, containing: 20 mM Hepes pH 7.5, 2 mM MgCl2, 5 mM KCl, 1 mM DTT, and protease inhibitor cocktail (Roche, Israel), supplemented with energy regeneration mixture (1 mM ATP, 7.5 mM creatine phosphate, 70 mg/ml creatine phosphokinase, 0.1 mM EGTA). Cells were incubated in swelling buffer on ice for 30 min and homogenized by freeze-thawing in liquid nitrogen and passage through a 21 G needle. Extracts were cleared by subsequent centrifugations (14,000 RPM, 10 min; 14,000 RPM for 40 min), quick frozen in liquid nitrogen, and stored at −80 °C.

### Ligand-dependent c-Kit autophosphorylation assay

Double-tagged proteins were expressed off chip using linear DNA and TNT T7 mix, in the presence or absence of microsomal membranes. After expression, the proteins were incubated with different concentrations of Stem Cell Factor (SCF), for 30 min at RT and were then immobilized under the button via anti-Myc-biotinylated antibody. Next, a 10 min wash with PBS buffer was performed. Protein expression levels were evaluated following incubation (30 min) with anti-His-Alexa-Fluor 633 antibody (Qiagen, Venlo, Netherlands) and wash (10 min). Autophosphorylation levels were determined following incubation (30 min) with anti-P-Tyr fluorescently labeled with Alexa-Fluor 647 or Alexa-Fluor 488 (Cell Signaling, Danvers, MA, USA) and a wash (10 min). Net autophosphorylation levels were normalize to protein expression levels.

## Supplementary information

Supplementary Information

## Data Availability

The data that support the findings of this study are attached as source data.
