# Supplementary material for: A high-throughput integrated microfluidics method enables tyrosine autophosphorylation discovery
Source: Commun Biol. 2019 Jan 30;2:42. doi: 10.1038/s42003-019-0286-9 (PMC6353932; doi:10.1038/s42003-019-0286-9)
Supplement: Supplementary file 1 — Supplementary Information [file 42003_2019_286_MOESM1_ESM.pdf]

Supplementary Figures and supplementary data Figures and tables.

Supplementary Figure 1

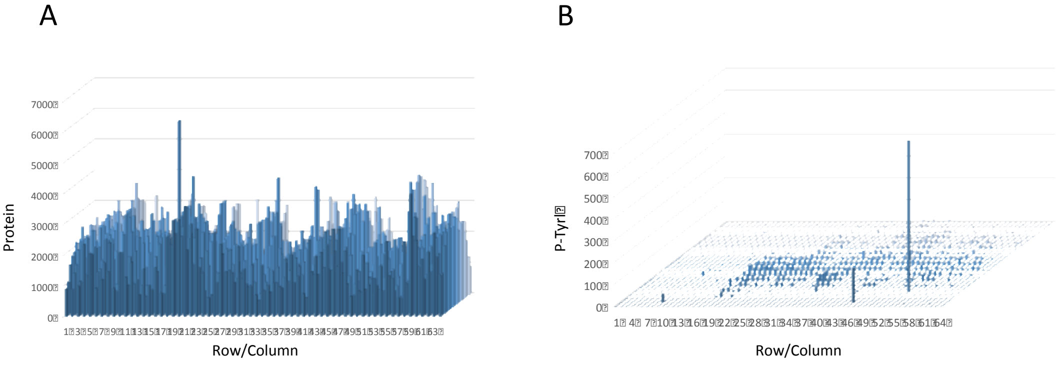

Supplementary Figure 1. ***Pivot table of protein array.*** An ORF library of 882 human proteins double-tagged with His and Myc sequences was printed in quadruplicates for on-chip expression and immobilization via His tag. The level of the arrayed proteins was determined *in situ* with Cy3-coupled anti-Myc antibodies (A) while P-Tyr signal was determined with Cy5-coupled anti P-Tyr antibodies (B). Raw images are available in tiff format. Average values are plotted (n=4). Fluorescence levels are provided in arbitrary units. Source data provided in Supplementary Data of Figure 2A.

Source Data - Supplementary Figure 2

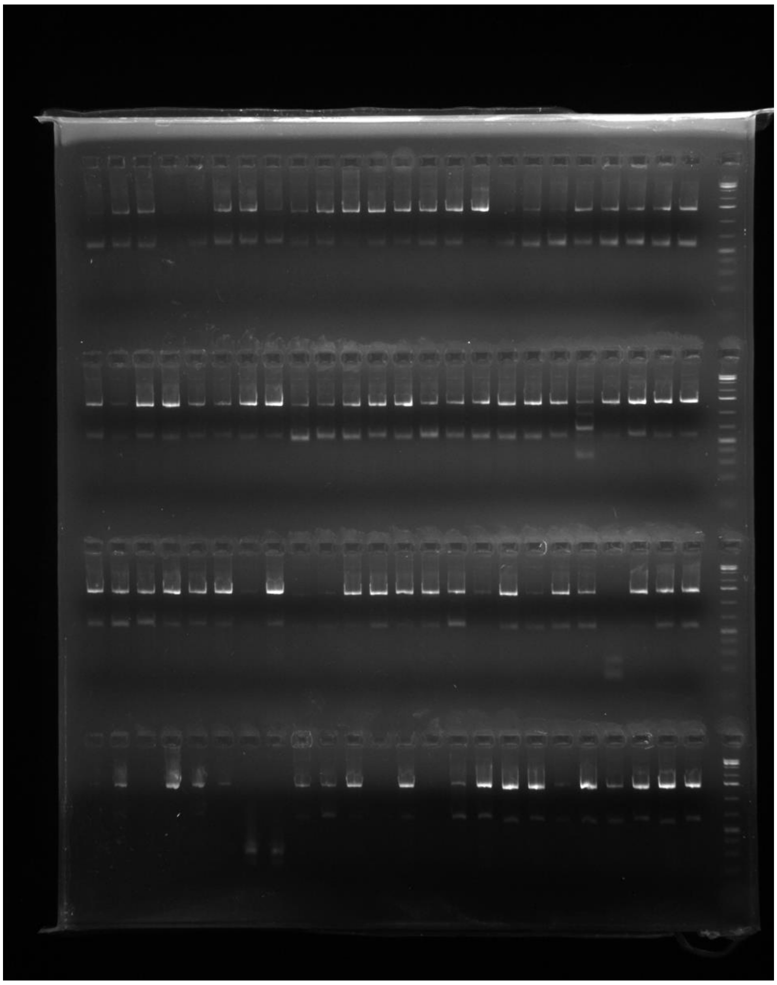

Supplementary Figure 2. **Source data for agarose gel presenting the full array of human PCR products, to be later print on the microfluidic device.**

Supplementary Figure 3

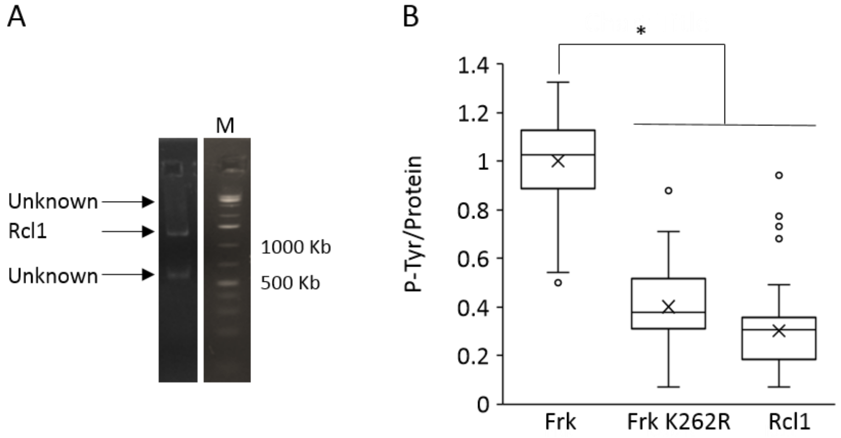

Supplementary Figure 3 .A validation for Rcl1 possible autophosphorylation. A. Image extracted from original gels used for validation of the PCR assembly of the library. It is possible to see that the Rcl1 PCR assembly reaction was contaminated with unknown DNA. Source data provided in Supplementary Data of Figure 2A B. We isolated the Rcl1 cDNA and tested it for activity in comparison with wt and kinase-dead Frk (negative control). Results were normalized to each protein expression level (P-Tyr/Protein), as well as to maximum activity (Frk phosphorylation level) .Proteins were immobilized by anti His biotin antibody. Proteins expression level was evaluated using anti C-myc. Autophosphorylation level was evaluated by anti P-Tyr. (n=64, (*) $p<0.001$). No autophosphorylation was observed for Rcl1.

Source Data - Supplementary Figure 4

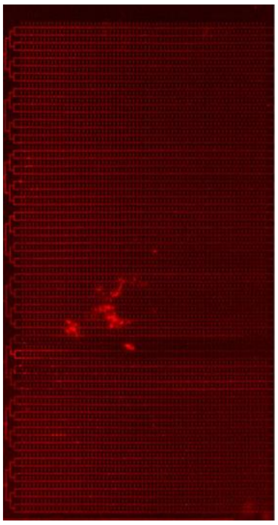

Supplementary Figure 4. **Source data for chip screening of Hck, Hck K290E and Securin phosphorylation.**

Source Data - Supplementary Figure 5

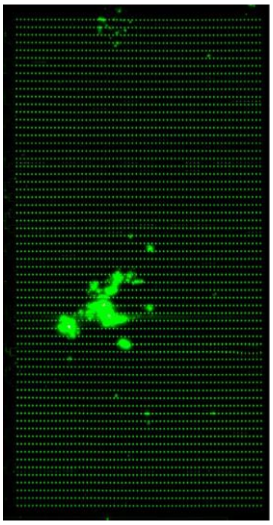

Supplementary Figure 5. **Source data for chip screening of Hck, Hck K290E and Securin expression.**

Source Data - Supplementary Figure 6

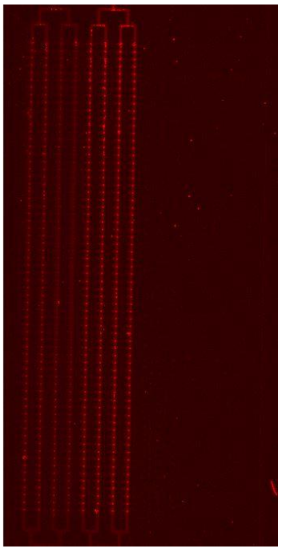

Supplementary Figure 6. **Source data for chip screening of Frk and Frk K262R on chip immunoprecipitation.**

Source Data - Supplementary Figure 7

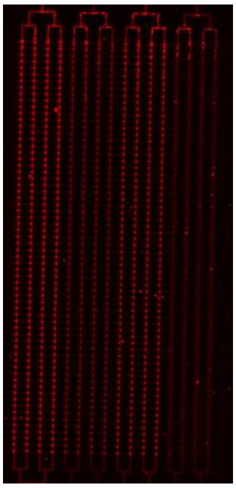

Supplementary Figure 7. Source data for chip screening of Hck and Hck K90E on chip immunoprecipitation

Source Data - Supplementary Figure 8

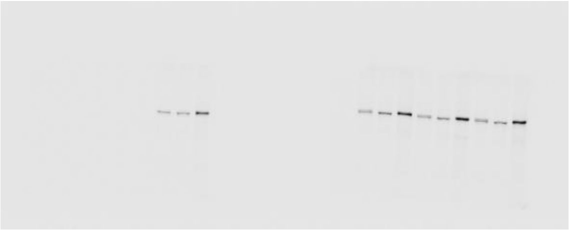

Supplementary Figure 8. **Source data for mobility shift assay of Frk.**

Source Data - Supplementary Figure 9

Supplementary Figure 9. **Source data for mobility shift assay of Hck.**

Source Data - Supplementary Figure 10

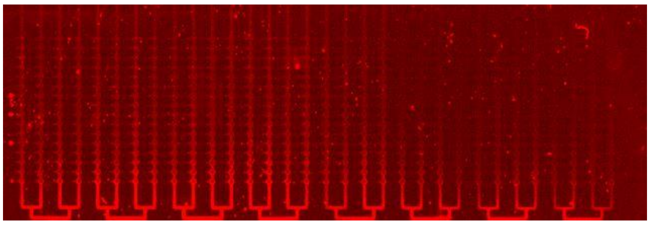

Supplementary Figure 10. **Source data for chip screening of Fgfr1, Fgfr1 K512R and Securin phosphorylation.**

Source Data - Supplementary Figure 11

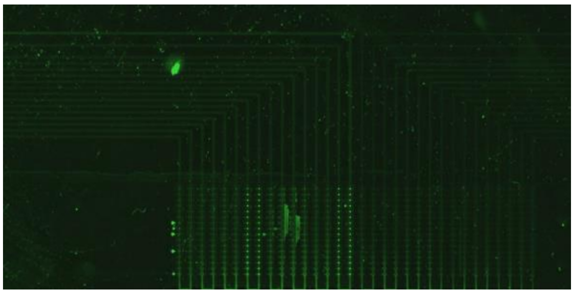

Supplementary Figure 11. **Source data for chip screening of Fgfr1, Fgfr1 K512R and Securin expression.**

Source Data - Supplementary Figure 12

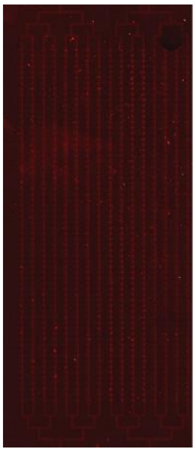

Supplementary Figure 12. Source data for chip screening of Fgfr1 and Fgfr1 K512R immunoprecipitation on chip

Supplementary Figure 13

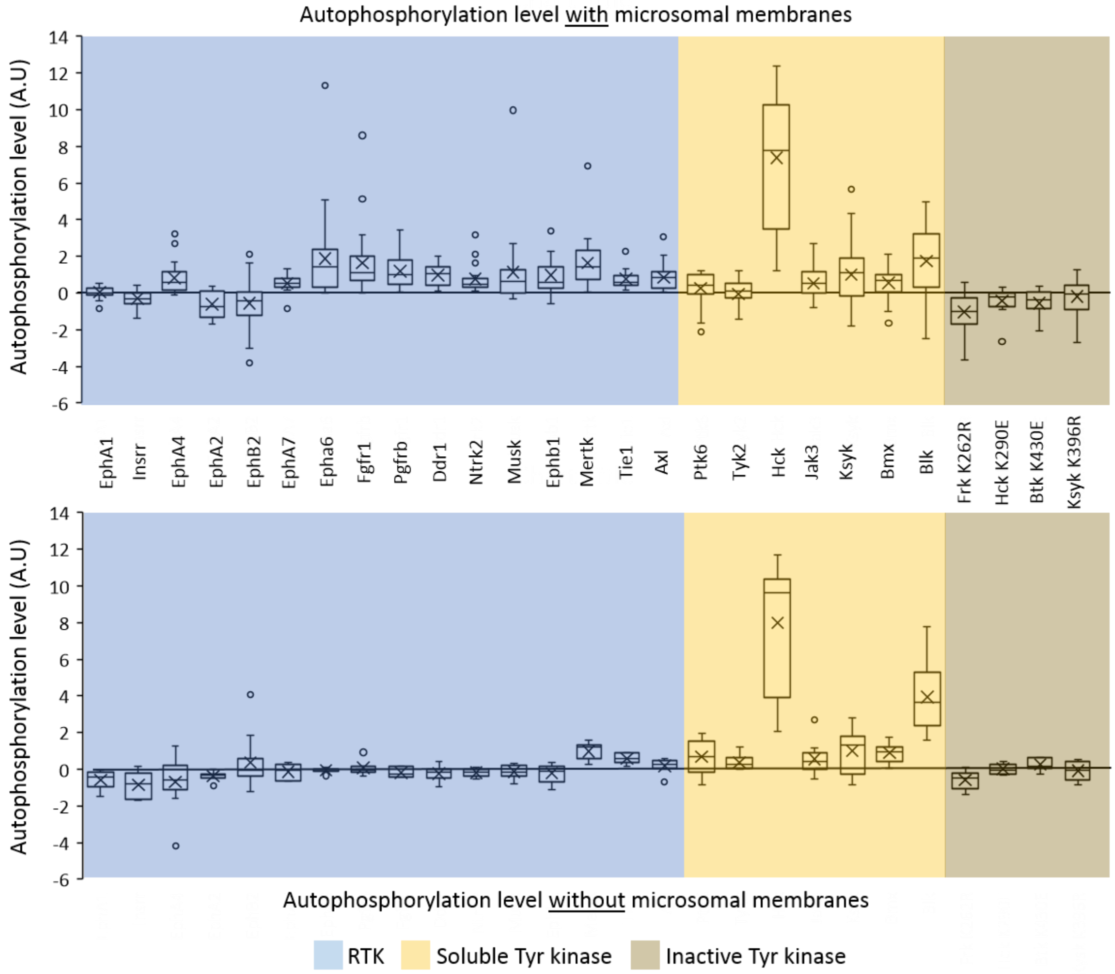

Supplementary Figure 13. ***On chip autophosphorylation of receptor Tyr kinases (RTKs) in membranous or aqueous environments.*** An ORF library comprising 17 RTKs, 7 soluble Tyr kinases, and 4 inactive Tyr kinases was spotted on chip in quadruplicates. Proteins were expressed in reticulocyte lysate supplemented with microsomal membranes or mock. Mean P-Tyr signal derived from 3 independent experiments were determined for the arrayed proteins, and plotted following subtraction of non-specific background signal, i.e., mean P-Tyr signal measured for inactive kinase mutants. (n=15-31). Error bars = 1 SE.

Source Data - Supplementary Figure 14

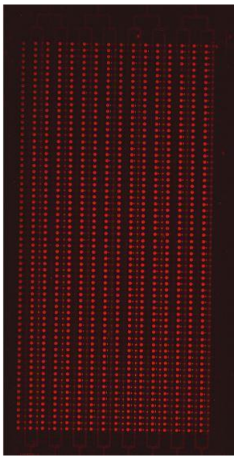

Supplementary Figure 14. **Source data for chip screening of Ror2 and Ror2 K507E phosphorylation.**

Supplementary Figure 15

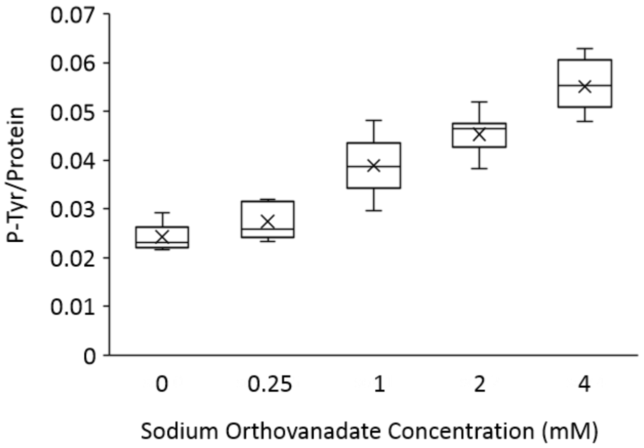

Supplementary Figure 15. Sodium Orthovanadate dose response of Frk. Frk was immobilized on chip using anti His biotin antibody and incubated for 30 min, 37°c in parallel with HEK293 cell extracts supplemented with increasing amounts of Sodium Orthovanadate. Frk was immuno-labeled on-chip with Cy5-coupled anti-P-Tyr antibodies (n=10).

Supplementary Figure 16

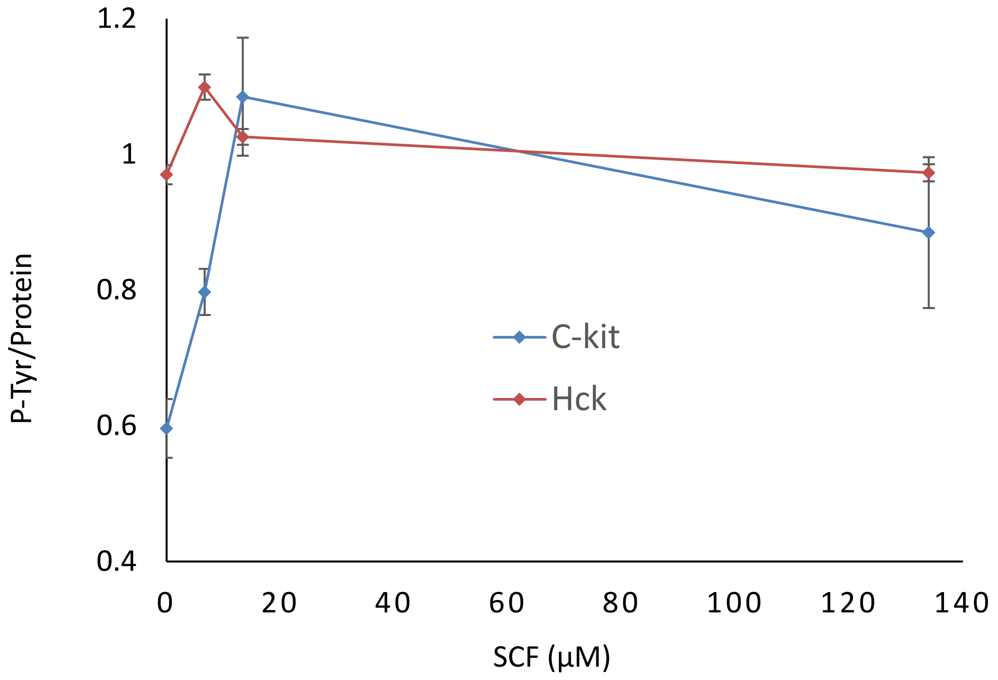

Supplementary Figure 16. *On-chip detection of ligand-dependent autophosphorylation.* Myc/His-tagged C-Kit was expressed in reticulocyte lysate supplemented with microsomal membranes. Protein products were then incubated (30 min; RT) with increasing concentrations of stem cell factor (SCF), and immobilized to protein chambers for an on-chip autophosphorylation assay. Average P-Tyr to protein ratios were determined (n=13). Data is normalized to maximal activity. A control experiment with Hck was performed in a similar manner. Error bars = 1 SE.

Supplementary Figure 17

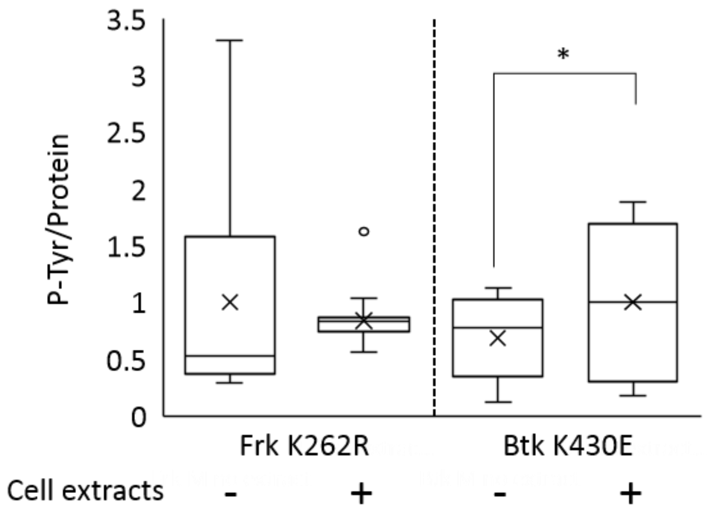

Supplementary Figure. 17. ***Distinguishing phosphorylation from autophosphorylation activity on chip.*** Myc/His-tagged inactive Frk and Btk mutants were expressed in reticulocyte lysate, immobilized to protein chambers, and incubated on-chip with active HEK293 cell extracts (30 min; 37°C). Average P-Tyr to protein ratios were determined (*n*=21-24, (*) P<0.05). Data is normalized to maximal activity. Error bars = 1 SE.

Supplementary Table 1: BC numbers and on chip location of the human arrayed proteins.

Protein	BC number	Column	Row
SUV92	BC007754	1	1
PDK2	BC005811	1	2
ZN165	BC026092	1	3
LN28A	BC028566	1	4
BARX2	BC111432	1	5
PIAS2	BC015190	1	6
WBP11	BC001621	1	7
T2H2L	BC064557	1	8
RCL1	BC001025	1	9
STRAB	BC008302	1	10
ZN689	BC014000	1	11
METL8	BC025250	1	12
PSPC1	BC014184	1	13
DTBP1	BC011912	1	14
GNL3	BC001024	1	15
RHXF1	BC069324	1	16
ZN830	BC011584	1	17
TYY1	BC037308	1	18
HSBP1	BC007515	1	19
CENPS	BC029430	1	20
TF2LY	BC128604	1	21
CHD2	BC007347	1	22
KLF11	BC063286	1	23
DCNP1	BC069608	1	24
PER3	BC026102	1	25
BRE	BC001251	1	26
DPOE3	BC003166	1	27
DEDD2	BC013372	1	28
HNRPQ	BC032643	1	29
PPIL2	BC028385	1	30
DYRK3	BC015501	1	31
SYUA	BC013293	1	32
ANXA1	BC001275	1	33
U2AF2	BC008740	1	34
DMRTB	BC029566	1	35
CRTC1	BC028050	1	37
AP2A	BC017754	1	38
RBY1A	BC047768	1	39
AP1AR	BC009485	1	40
Z385D	BC007212	1	41
AL1A2	BC030589	1	42

THOC7	BC065012	1	43
H2B1B	BC096728	1	44
PESC	BC032489	1	45
FRK	BC012916	1	46
CREB5	BC059400	1	47
RSRC1	BC006982	1	48
CDCA7	BC027966	1	49
ZN563	BC022523	1	50
APC10	BC005217	1	51
ZKSC4	BC014031	1	53
NOP10	BC008886	1	54
FAP24	BC003535	1	55
RRAGC	BC016668	1	57
MEF2A	BC013437	1	58
PHF19	BC022374	1	59
H14	BC096168	1	60
MUS81	BC009999	1	61
ZN551	BC005868	1	62
ATPF2	BC004114	1	63
SOX7	BC071947	1	64
SUV92	BC007754	2	1
PDK2	BC005811	2	2
ZN165	BC026092	2	3
LN28A	BC028566	2	4
BARX2	BC111432	2	5
PIAS2	BC015190	2	6
WBP11	BC001621	2	7
T2H2L	BC064557	2	8
RCL1	BC001025	2	9
STRAB	BC008302	2	10
ZN689	BC014000	2	11
METL8	BC025250	2	12
PSPC1	BC014184	2	13
DTBP1	BC011912	2	14
GNL3	BC001024	2	15
RHXF1	BC069324	2	16
ZN830	BC011584	2	17
TYY1	BC037308	2	18
HSBP1	BC007515	2	19
CENPS	BC029430	2	20
TF2LY	BC128604	2	21
CHD2	BC007347	2	22
KLF11	BC063286	2	23
DCNP1	BC069608	2	24
PER3	BC026102	2	25
BRE	BC001251	2	26

DPOE3	BC003166	2	27
DEDD2	BC013372	2	28
HNRPQ	BC032643	2	29
PPIL2	BC028385	2	30
DYRK3	BC015501	2	31
SYUA	BC013293	2	32
ANXA1	BC001275	2	33
U2AF2	BC008740	2	34
DMRTB	BC029566	2	35
CRTC1	BC028050	2	37
AP2A	BC017754	2	38
RBY1A	BC047768	2	39
AP1AR	BC009485	2	40
Z385D	BC007212	2	41
AL1A2	BC030589	2	42
THOC7	BC065012	2	43
H2B1B	BC096728	2	44
PESC	BC032489	2	45
FRK	BC012916	2	46
CREB5	BC059400	2	47
RSRC1	BC006982	2	48
CDCA7	BC027966	2	49
ZN563	BC022523	2	50
APC10	BC005217	2	51
ZKSC4	BC014031	2	53
NOP10	BC008886	2	54
FAP24	BC003535	2	55
RRAGC	BC016668	2	57
MEF2A	BC013437	2	58
PHF19	BC022374	2	59
H14	BC096168	2	60
MUS81	BC009999	2	61
ZN551	BC005868	2	62
ATPF2	BC004114	2	63
SOX7	BC071947	2	64
SUV92	BC007754	3	1
PDK2	BC005811	3	2
ZN165	BC026092	3	3
LN28A	BC028566	3	4
BARX2	BC111432	3	5
PIAS2	BC015190	3	6
WBP11	BC001621	3	7
T2H2L	BC064557	3	8
RCL1	BC001025	3	9
STRAB	BC008302	3	10
ZN689	BC014000	3	11

METL8	BC025250	3	12
PSPC1	BC014184	3	13
DTBP1	BC011912	3	14
GNL3	BC001024	3	15
RHXF1	BC069324	3	16
ZN830	BC011584	3	17
TYY1	BC037308	3	18
HSBP1	BC007515	3	19
CENPS	BC029430	3	20
TF2LY	BC128604	3	21
CHD2	BC007347	3	22
KLF11	BC063286	3	23
DCNP1	BC069608	3	24
PER3	BC026102	3	25
BRE	BC001251	3	26
DPOE3	BC003166	3	27
DEDD2	BC013372	3	28
HNRPQ	BC032643	3	29
PPIL2	BC028385	3	30
DYRK3	BC015501	3	31
SYUA	BC013293	3	32
ANXA1	BC001275	3	33
U2AF2	BC008740	3	34
DMRTB	BC029566	3	35
CRTC1	BC028050	3	37
AP2A	BC017754	3	38
RBY1A	BC047768	3	39
AP1AR	BC009485	3	40
Z385D	BC007212	3	41
AL1A2	BC030589	3	42
THOC7	BC065012	3	43
H2B1B	BC096728	3	44
PESC	BC032489	3	45
FRK	BC012916	3	46
CREB5	BC059400	3	47
RSRC1	BC006982	3	48
CDCA7	BC027966	3	49
ZN563	BC022523	3	50
APC10	BC005217	3	51
ZKSC4	BC014031	3	53
NOP10	BC008886	3	54
FAP24	BC003535	3	55
RRAGC	BC016668	3	57
MEF2A	BC013437	3	58
PHF19	BC022374	3	59
H14	BC096168	3	60

MUS81	BC009999	3	61
ZN551	BC005868	3	62
ATPF2	BC004114	3	63
SOX7	BC071947	3	64
SUV92	BC007754	4	1
PDK2	BC005811	4	2
ZN165	BC026092	4	3
LN28A	BC028566	4	4
BARX2	BC111432	4	5
PIAS2	BC015190	4	6
WBP11	BC001621	4	7
T2H2L	BC064557	4	8
RCL1	BC001025	4	9
STRAB	BC008302	4	10
ZN689	BC014000	4	11
METL8	BC025250	4	12
PSPC1	BC014184	4	13
DTBP1	BC011912	4	14
GNL3	BC001024	4	15
RHXF1	BC069324	4	16
ZN830	BC011584	4	17
TYY1	BC037308	4	18
HSBP1	BC007515	4	19
CENPS	BC029430	4	20
TF2LY	BC128604	4	21
CHD2	BC007347	4	22
KLF11	BC063286	4	23
DCNP1	BC069608	4	24
PER3	BC026102	4	25
BRE	BC001251	4	26
DPOE3	BC003166	4	27
DEDD2	BC013372	4	28
HNRPQ	BC032643	4	29
PPIL2	BC028385	4	30
DYRK3	BC015501	4	31
SYUA	BC013293	4	32
ANXA1	BC001275	4	33
U2AF2	BC008740	4	34
DMRTB	BC029566	4	35
CRTC1	BC028050	4	37
AP2A	BC017754	4	38
RBY1A	BC047768	4	39
AP1AR	BC009485	4	40
Z385D	BC007212	4	41
AL1A2	BC030589	4	42
THOC7	BC065012	4	43

H2B1B	BC096728	4	44
PESC	BC032489	4	45
FRK	BC012916	4	46
CREB5	BC059400	4	47
RSRC1	BC006982	4	48
CDCA7	BC027966	4	49
ZN563	BC022523	4	50
APC10	BC005217	4	51
ZKSC4	BC014031	4	53
NOP10	BC008886	4	54
FAP24	BC003535	4	55
RRAGC	BC016668	4	57
MEF2A	BC013437	4	58
PHF19	BC022374	4	59
H14	BC096168	4	60
MUS81	BC009999	4	61
ZN551	BC005868	4	62
ATPF2	BC004114	4	63
SOX7	BC071947	4	64
DUS11	BC000346	5	1
AGO2	BC007633	5	2
NUF2	BC008489	5	3
ANR49	BC017798	5	4
VPS4B	BC039574	5	7
DD19B	BC010008	5	8
CDK7	BC005298	5	9
ARFG1	BC028233	5	10
RXRG	BC012063	5	11
PSA3	BC029402	5	12
MTA3	BC004227	5	13
RABL6	BC002945	5	14
AKP8L	BC000713	5	15
FOSL2	BC022791	5	16
ATX3	BC033711	5	17
LHX4	BC011759	5	18
S100B	BC001766	5	19
ZN695	BC023527	5	20
RPA49	BC014331	5	21
PRP19	BC008719	5	22
ZN449	BC060768	5	23
RFOX2	BC025281	5	24
TISB	BC018340	5	25
RPGF5	BC039203	5	26
PTN22	BC017785	5	28
F214B	BC004406	5	29
ZN238	BC036677	5	30

RSSA	BC050688	5	31
CNTF	BC068030	5	32
TF7L2	BC031056	5	33
SSF1	BC009833	5	34
MUSC	BC006313	5	35
LBH	BC126434	5	36
PSMD3	BC012302	5	37
GSK3B	BC000251	5	38
CEBPE	BC035797	5	39
MEMO1	BC070046	5	40
ZN821	BC012116	5	41
DCA13	BC026067	5	42
ERGI2	BC064522	5	43
H31	BC096128	5	44
CSTFT	BC028239	5	45
ZN273	BC019234	5	46
LEF1	BC050632	5	47
PSD12	BC065826	5	48
DUS4	BC002671	5	49
ST38L	BC028603	5	50
FRG1	BC053997	5	51
FER3L	BC101135	5	52
P4R3A	BC038932	5	53
STK33	BC031231	5	54
MS18A	BC042917	5	55
SRBS2	BC011883	5	56
RBM4	BC032735	5	57
RARA	BC008727	5	58
H31	BC031333	5	59
HXB1	BC096192	5	60
EME1	BC016470	5	61
HERC6	BC035775	5	62
P2R3C	BC063438	5	63
CREST	BC068993	5	64
DUS11	BC000346	6	1
AGO2	BC007633	6	2
NUF2	BC008489	6	3
ANR49	BC017798	6	4
VPS4B	BC039574	6	7
DD19B	BC010008	6	8
CDK7	BC005298	6	9
ARFG1	BC028233	6	10
RXRG	BC012063	6	11
PSA3	BC029402	6	12
MTA3	BC004227	6	13
RABL6	BC002945	6	14

AKP8L	BC000713	6	15
FOSL2	BC022791	6	16
ATX3	BC033711	6	17
LHX4	BC011759	6	18
S100B	BC001766	6	19
ZN695	BC023527	6	20
RPA49	BC014331	6	21
PRP19	BC008719	6	22
ZN449	BC060768	6	23
RFOX2	BC025281	6	24
TISB	BC018340	6	25
RPGF5	BC039203	6	26
PTN22	BC017785	6	28
F214B	BC004406	6	29
ZN238	BC036677	6	30
RSSA	BC050688	6	31
CNTF	BC068030	6	32
TF7L2	BC031056	6	33
SSF1	BC009833	6	34
MUSC	BC006313	6	35
LBH	BC126434	6	36
PSMD3	BC012302	6	37
GSK3B	BC000251	6	38
CEBPE	BC035797	6	39
MEMO1	BC070046	6	40
ZN821	BC012116	6	41
DCA13	BC026067	6	42
ERGI2	BC064522	6	43
H31	BC096128	6	44
CSTFT	BC028239	6	45
ZN273	BC019234	6	46
LEF1	BC050632	6	47
PSD12	BC065826	6	48
DUS4	BC002671	6	49
ST38L	BC028603	6	50
FRG1	BC053997	6	51
FER3L	BC101135	6	52
P4R3A	BC038932	6	53
STK33	BC031231	6	54
MS18A	BC042917	6	55
SRBS2	BC011883	6	56
RBM4	BC032735	6	57
RARA	BC008727	6	58
H31	BC031333	6	59
HXB1	BC096192	6	60
EME1	BC016470	6	61

HERC6	BC035775	6	62
P2R3C	BC063438	6	63
CREST	BC068993	6	64
DUS11	BC000346	7	1
AGO2	BC007633	7	2
NUF2	BC008489	7	3
ANR49	BC017798	7	4
VPS4B	BC039574	7	7
DD19B	BC010008	7	8
CDK7	BC005298	7	9
ARFG1	BC028233	7	10
RXRG	BC012063	7	11
PSA3	BC029402	7	12
MTA3	BC004227	7	13
RABL6	BC002945	7	14
AKP8L	BC000713	7	15
FOSL2	BC022791	7	16
ATX3	BC033711	7	17
LHX4	BC011759	7	18
S100B	BC001766	7	19
ZN695	BC023527	7	20
RPA49	BC014331	7	21
PRP19	BC008719	7	22
ZN449	BC060768	7	23
RFOX2	BC025281	7	24
TISB	BC018340	7	25
RPGF5	BC039203	7	26
PTN22	BC017785	7	28
F214B	BC004406	7	29
ZN238	BC036677	7	30
RSSA	BC050688	7	31
CNTF	BC068030	7	32
TF7L2	BC031056	7	33
SSF1	BC009833	7	34
MUSC	BC006313	7	35
LBH	BC126434	7	36
PSMD3	BC012302	7	37
GSK3B	BC000251	7	38
CEBPE	BC035797	7	39
MEMO1	BC070046	7	40
ZN821	BC012116	7	41
DCA13	BC026067	7	42
ERGI2	BC064522	7	43
H31	BC096128	7	44
CSTFT	BC028239	7	45
ZN273	BC019234	7	46

LEF1	BC050632	7	47
PSD12	BC065826	7	48
DUS4	BC002671	7	49
ST38L	BC028603	7	50
FRG1	BC053997	7	51
FER3L	BC101135	7	52
P4R3A	BC038932	7	53
STK33	BC031231	7	54
MS18A	BC042917	7	55
SRBS2	BC011883	7	56
RBM4	BC032735	7	57
RARA	BC008727	7	58
H31	BC031333	7	59
HXB1	BC096192	7	60
EME1	BC016470	7	61
HERC6	BC035775	7	62
P2R3C	BC063438	7	63
CREST	BC068993	7	64
DUS11	BC000346	8	1
AGO2	BC007633	8	2
NUF2	BC008489	8	3
ANR49	BC017798	8	4
VPS4B	BC039574	8	7
DD19B	BC010008	8	8
CDK7	BC005298	8	9
ARFG1	BC028233	8	10
RXRG	BC012063	8	11
PSA3	BC029402	8	12
MTA3	BC004227	8	13
RABL6	BC002945	8	14
AKP8L	BC000713	8	15
FOSL2	BC022791	8	16
ATX3	BC033711	8	17
LHX4	BC011759	8	18
S100B	BC001766	8	19
ZN695	BC023527	8	20
RPA49	BC014331	8	21
PRP19	BC008719	8	22
ZN449	BC060768	8	23
RFOX2	BC025281	8	24
TISB	BC018340	8	25
RPGF5	BC039203	8	26
PTN22	BC017785	8	28
F214B	BC004406	8	29
ZN238	BC036677	8	30
RSSA	BC050688	8	31

CNTF	BC068030	8	32
TF7L2	BC031056	8	33
SSF1	BC009833	8	34
MUSC	BC006313	8	35
LBH	BC126434	8	36
PSMD3	BC012302	8	37
GSK3B	BC000251	8	38
CEBPE	BC035797	8	39
MEMO1	BC070046	8	40
ZN821	BC012116	8	41
DCA13	BC026067	8	42
ERGI2	BC064522	8	43
H31	BC096128	8	44
CSTFT	BC028239	8	45
ZN273	BC019234	8	46
LEF1	BC050632	8	47
PSD12	BC065826	8	48
DUS4	BC002671	8	49
ST38L	BC028603	8	50
FRG1	BC053997	8	51
FER3L	BC101135	8	52
P4R3A	BC038932	8	53
STK33	BC031231	8	54
MS18A	BC042917	8	55
SRBS2	BC011883	8	56
RBM4	BC032735	8	57
RARA	BC008727	8	58
H31	BC031333	8	59
HXB1	BC096192	8	60
EME1	BC016470	8	61
HERC6	BC035775	8	62
P2R3C	BC063438	8	63
CREST	BC068993	8	64
AFF4	BC025700	9	1
NR1H3	BC008819	9	2
TRI22	BC035582	9	3
GID8	BC032120	9	4
TBX20	BC120946	9	5
PHF13	BC038516	9	6
WAP53	BC002336	9	7
VGLL2	BC069316	9	8
PCBP4	BC017098	9	9
ZN566	BC007064	9	10
P66A	BC011684	9	11
RU2A	BC022816	9	12
CBX6	BC012111	9	13

SAS10	BC004546	9	14
ERG	BC040168	9	15
TDIF1	BC024290	9	16
TCF19	BC002493	9	17
THEG	BC028574	9	18
SUMO2	BC008450	9	19
CK001	BC020628	9	20
NCF2	BC001606	9	21
MIER2	BC028203	9	22
ENC1	BC000418	9	23
RFA4	BC069791	9	24
KHDR2	BC034043	9	25
SETD6	BC022451	9	26
NT5C	BC008183	9	27
COMD1	BC022046	9	28
RNF12	BC013357	9	29
SKP2	BC001441	9	30
KTNA1	BC050428	9	31
ZMYM5	BC007048	9	32
MDHM	BC001917	9	33
DUS10	BC031405	9	34
CSDC2	BC067113	9	35
H4	BC120939	9	36
UTP6	BC035325	9	37
STPAP	BC005013	9	38
RSSA	BC066941	9	39
LDB3	BC010929	9	40
RAD9A	BC014848	9	41
TFEB	BC032448	9	42
U2AF4	BC021186	9	43
H31	BC096132	9	44
RORA	BC008831	9	45
ETV5	BC007333	9	46
SMAD3	BC050743	9	47
SNR40	BC001494	9	48
DMRTD	BC039266	9	49
UFSP2	BC010493	9	50
ATF3	BC006322	9	51
MPIP1	BC007401	9	53
HCK	BC014435	9	54
STK16	BC053998	9	55
ZBT47	BC021855	9	57
NLE1	BC012075	9	58
BUD31	BC022821	9	59
CBS	BC010242	9	61
PCM1	BC000453	9	62

AFF4	BC063007	9	63
ZN595	BC036110	9	64
AFF4	BC025700	10	1
NR1H3	BC008819	10	2
TRI22	BC035582	10	3
GID8	BC032120	10	4
TBX20	BC120946	10	5
PHF13	BC038516	10	6
WAP53	BC002336	10	7
VGLL2	BC069316	10	8
PCBP4	BC017098	10	9
ZN566	BC007064	10	10
P66A	BC011684	10	11
RU2A	BC022816	10	12
CBX6	BC012111	10	13
SAS10	BC004546	10	14
ERG	BC040168	10	15
TDIF1	BC024290	10	16
TCF19	BC002493	10	17
THEG	BC028574	10	18
SUMO2	BC008450	10	19
CK001	BC020628	10	20
NCF2	BC001606	10	21
MIER2	BC028203	10	22
ENC1	BC000418	10	23
RFA4	BC069791	10	24
KHDR2	BC034043	10	25
SETD6	BC022451	10	26
NT5C	BC008183	10	27
COMD1	BC022046	10	28
RNF12	BC013357	10	29
SKP2	BC001441	10	30
KTNA1	BC050428	10	31
ZMYM5	BC007048	10	32
MDHM	BC001917	10	33
DUS10	BC031405	10	34
CSDC2	BC067113	10	35
H4	BC120939	10	36
UTP6	BC035325	10	37
STPAP	BC005013	10	38
RSSA	BC066941	10	39
LDB3	BC010929	10	40
RAD9A	BC014848	10	41
TFEB	BC032448	10	42
U2AF4	BC021186	10	43
H31	BC096132	10	44

RORA	BC008831	10	45
ETV5	BC007333	10	46
SMAD3	BC050743	10	47
SNR40	BC001494	10	48
DMRTD	BC039266	10	49
UFSP2	BC010493	10	50
ATF3	BC006322	10	51
MPIP1	BC007401	10	53
HCK	BC014435	10	54
STK16	BC053998	10	55
ZBT47	BC021855	10	57
NLE1	BC012075	10	58
BUD31	BC022821	10	59
CBS	BC010242	10	61
PCM1	BC000453	10	62
AFF4	BC063007	10	63
ZN595	BC036110	10	64
AFF4	BC025700	11	1
NR1H3	BC008819	11	2
TRI22	BC035582	11	3
GID8	BC032120	11	4
TBX20	BC120946	11	5
PHF13	BC038516	11	6
WAP53	BC002336	11	7
VGLL2	BC069316	11	8
PCBP4	BC017098	11	9
ZN566	BC007064	11	10
P66A	BC011684	11	11
RU2A	BC022816	11	12
CBX6	BC012111	11	13
SAS10	BC004546	11	14
ERG	BC040168	11	15
TDIF1	BC024290	11	16
TCF19	BC002493	11	17
THEG	BC028574	11	18
SUMO2	BC008450	11	19
CK001	BC020628	11	20
NCF2	BC001606	11	21
MIER2	BC028203	11	22
ENC1	BC000418	11	23
RFA4	BC069791	11	24
KHDR2	BC034043	11	25
SETD6	BC022451	11	26
NT5C	BC008183	11	27
COMD1	BC022046	11	28
RNF12	BC013357	11	29

SKP2	BC001441	11	30
KTNA1	BC050428	11	31
ZMYM5	BC007048	11	32
MDHM	BC001917	11	33
DUS10	BC031405	11	34
CSDC2	BC067113	11	35
H4	BC120939	11	36
UTP6	BC035325	11	37
STPAP	BC005013	11	38
RSSA	BC066941	11	39
LDB3	BC010929	11	40
RAD9A	BC014848	11	41
TFEB	BC032448	11	42
U2AF4	BC021186	11	43
H31	BC096132	11	44
RORA	BC008831	11	45
ETV5	BC007333	11	46
SMAD3	BC050743	11	47
SNR40	BC001494	11	48
DMRTD	BC039266	11	49
UFSP2	BC010493	11	50
ATF3	BC006322	11	51
MPIP1	BC007401	11	53
HCK	BC014435	11	54
STK16	BC053998	11	55
ZBT47	BC021855	11	57
NLE1	BC012075	11	58
BUD31	BC022821	11	59
CBS	BC010242	11	61
PCM1	BC000453	11	62
AFF4	BC063007	11	63
ZN595	BC036110	11	64
AFF4	BC025700	12	1
NR1H3	BC008819	12	2
TRI22	BC035582	12	3
GID8	BC032120	12	4
TBX20	BC120946	12	5
PHF13	BC038516	12	6
WAP53	BC002336	12	7
VGLL2	BC069316	12	8
PCBP4	BC017098	12	9
ZN566	BC007064	12	10
P66A	BC011684	12	11
RU2A	BC022816	12	12
CBX6	BC012111	12	13
SAS10	BC004546	12	14

ERG	BC040168	12	15
TDIF1	BC024290	12	16
TCF19	BC002493	12	17
THEG	BC028574	12	18
SUMO2	BC008450	12	19
CK001	BC020628	12	20
NCF2	BC001606	12	21
MIER2	BC028203	12	22
ENC1	BC000418	12	23
RFA4	BC069791	12	24
KHDR2	BC034043	12	25
SETD6	BC022451	12	26
NT5C	BC008183	12	27
COMD1	BC022046	12	28
RNF12	BC013357	12	29
SKP2	BC001441	12	30
KTNA1	BC050428	12	31
ZMYM5	BC007048	12	32
MDHM	BC001917	12	33
DUS10	BC031405	12	34
CSDC2	BC067113	12	35
H4	BC120939	12	36
UTP6	BC035325	12	37
STPAP	BC005013	12	38
RSSA	BC066941	12	39
LDB3	BC010929	12	40
RAD9A	BC014848	12	41
TFEB	BC032448	12	42
U2AF4	BC021186	12	43
H31	BC096132	12	44
RORA	BC008831	12	45
ETV5	BC007333	12	46
SMAD3	BC050743	12	47
SNR40	BC001494	12	48
DMRTD	BC039266	12	49
UFSP2	BC010493	12	50
ATF3	BC006322	12	51
MPIP1	BC007401	12	53
HCK	BC014435	12	54
STK16	BC053998	12	55
ZBT47	BC021855	12	57
NLE1	BC012075	12	58
BUD31	BC022821	12	59
CBS	BC010242	12	61
PCM1	BC000453	12	62
AFF4	BC063007	12	63

ZN595	BC036110	12	64
GBB2	BC012348	13	1
KC1D	BC003558	13	2
SIN1	BC002326	13	3
MYF6	BC017834	13	4
NR1I2	BC017304	13	5
FANCC	BC015748	13	6
PIAS3	BC001154	13	7
ATPF2	BC032126	13	8
RRS1	BC001811	13	9
KRR1	BC033887	13	10
ZN596	BC026190	13	11
R51A1	BC005973	13	12
IRF4	BC015752	13	13
PPIG	BC001555	13	14
SRS11	BC040436	13	15
NEIL2	BC013952	13	16
CAPG	BC000728	13	17
ZN670	BC005360	13	18
RL36	BC004971	13	19
ZN776	BC041615	13	20
ASCL4	BC128211	13	21
SMU1	BC002876	13	22
RPC5	BC000285	13	23
MTER3	BC025984	13	24
DCUP	BC001778	13	25
TULP3	BC032587	13	26
IF5A2	BC036072	13	27
KLDC3	BC012987	13	28
PNKP	BC033822	13	29
STAM2	BC028740	13	30
PEG10	BC050659	13	31
NEK2	BC065932	13	32
SNX15	BC009897	13	33
EIF3E	BC008419	13	34
ASGL1	BC064963	13	35
H2AB2	BC101415	13	36
ELF1	BC030507	13	37
NUSAP	BC024772	13	38
FANCL	BC054517	13	39
NPM	BC012566	13	40
ZN207	BC008023	13	41
BCL10	BC053617	13	43
ZNF34	BC004480	13	45
PUF60	BC011265	13	46
ZN410	BC050683	13	47

TRIB3	BC019363	13	48
SYTL2	BC015540	13	49
LARP6	BC006082	13	50
NAA20	BC005181	13	51
TBRG1	BC109269	13	52
PARP3	BC014260	13	53
PSMD5	BC014478	13	54
SIR6	BC004218	13	55
CN37	BC011046	13	57
PPIL4	BC020986	13	58
AN32A	BC125143	13	60
OPTN	BC032762	13	61
EIF3L	BC001101	13	62
DSN1	BC058899	13	63
BHE40	BC082238	13	64
GBB2	BC012348	14	1
KC1D	BC003558	14	2
SIN1	BC002326	14	3
MYF6	BC017834	14	4
NR1I2	BC017304	14	5
FANCC	BC015748	14	6
PIAS3	BC001154	14	7
ATPF2	BC032126	14	8
RRS1	BC001811	14	9
KRR1	BC033887	14	10
ZN596	BC026190	14	11
R51A1	BC005973	14	12
IRF4	BC015752	14	13
PPIG	BC001555	14	14
SRS11	BC040436	14	15
NEIL2	BC013952	14	16
CAPG	BC000728	14	17
ZN670	BC005360	14	18
RL36	BC004971	14	19
ZN776	BC041615	14	20
ASCL4	BC128211	14	21
SMU1	BC002876	14	22
RPC5	BC000285	14	23
MTER3	BC025984	14	24
DCUP	BC001778	14	25
TULP3	BC032587	14	26
IF5A2	BC036072	14	27
KLDC3	BC012987	14	28
PNKP	BC033822	14	29
STAM2	BC028740	14	30
PEG10	BC050659	14	31

NEK2	BC065932	14	32
SNX15	BC009897	14	33
EIF3E	BC008419	14	34
ASGL1	BC064963	14	35
H2AB2	BC101415	14	36
ELF1	BC030507	14	37
NUSAP	BC024772	14	38
FANCL	BC054517	14	39
NPM	BC012566	14	40
ZN207	BC008023	14	41
BCL10	BC053617	14	43
ZNF34	BC004480	14	45
PUF60	BC011265	14	46
ZN410	BC050683	14	47
TRIB3	BC019363	14	48
SYTL2	BC015540	14	49
LARP6	BC006082	14	50
NAA20	BC005181	14	51
TBRG1	BC109269	14	52
PARP3	BC014260	14	53
PSMD5	BC014478	14	54
SIR6	BC004218	14	55
CN37	BC011046	14	57
PPIL4	BC020986	14	58
AN32A	BC125143	14	60
OPTN	BC032762	14	61
EIF3L	BC001101	14	62
DSN1	BC058899	14	63
BHE40	BC082238	14	64
GBB2	BC012348	15	1
KC1D	BC003558	15	2
SIN1	BC002326	15	3
MYF6	BC017834	15	4
NR1I2	BC017304	15	5
FANCC	BC015748	15	6
PIAS3	BC001154	15	7
ATPF2	BC032126	15	8
RRS1	BC001811	15	9
KRR1	BC033887	15	10
ZN596	BC026190	15	11
R51A1	BC005973	15	12
IRF4	BC015752	15	13
PPIG	BC001555	15	14
SRS11	BC040436	15	15
NEIL2	BC013952	15	16
CAPG	BC000728	15	17

ZN670	BC005360	15	18
RL36	BC004971	15	19
ZN776	BC041615	15	20
ASCL4	BC128211	15	21
SMU1	BC002876	15	22
RPC5	BC000285	15	23
MTER3	BC025984	15	24
DCUP	BC001778	15	25
TULP3	BC032587	15	26
IF5A2	BC036072	15	27
KLDC3	BC012987	15	28
PNKP	BC033822	15	29
STAM2	BC028740	15	30
PEG10	BC050659	15	31
NEK2	BC065932	15	32
SNX15	BC009897	15	33
EIF3E	BC008419	15	34
ASGL1	BC064963	15	35
H2AB2	BC101415	15	36
ELF1	BC030507	15	37
NUSAP	BC024772	15	38
FANCL	BC054517	15	39
NPM	BC012566	15	40
ZN207	BC008023	15	41
BCL10	BC053617	15	43
ZNF34	BC004480	15	45
PUF60	BC011265	15	46
ZN410	BC050683	15	47
TRIB3	BC019363	15	48
SYTL2	BC015540	15	49
LARP6	BC006082	15	50
NAA20	BC005181	15	51
TBRG1	BC109269	15	52
PARP3	BC014260	15	53
PSMD5	BC014478	15	54
SIR6	BC004218	15	55
CN37	BC011046	15	57
PPIL4	BC020986	15	58
AN32A	BC125143	15	60
OPTN	BC032762	15	61
EIF3L	BC001101	15	62
DSN1	BC058899	15	63
BHE40	BC082238	15	64
GBB2	BC012348	16	1
KC1D	BC003558	16	2
SIN1	BC002326	16	3

MYF6	BC017834	16	4
NR1I2	BC017304	16	5
FANCC	BC015748	16	6
PIAS3	BC001154	16	7
ATPF2	BC032126	16	8
RRS1	BC001811	16	9
KRR1	BC033887	16	10
ZN596	BC026190	16	11
R51A1	BC005973	16	12
IRF4	BC015752	16	13
PPIG	BC001555	16	14
SRS11	BC040436	16	15
NEIL2	BC013952	16	16
CAPG	BC000728	16	17
ZN670	BC005360	16	18
RL36	BC004971	16	19
ZN776	BC041615	16	20
ASCL4	BC128211	16	21
SMU1	BC002876	16	22
RPC5	BC000285	16	23
MTER3	BC025984	16	24
DCUP	BC001778	16	25
TULP3	BC032587	16	26
IF5A2	BC036072	16	27
KLDC3	BC012987	16	28
PNKP	BC033822	16	29
STAM2	BC028740	16	30
PEG10	BC050659	16	31
NEK2	BC065932	16	32
SNX15	BC009897	16	33
EIF3E	BC008419	16	34
ASGL1	BC064963	16	35
H2AB2	BC101415	16	36
ELF1	BC030507	16	37
NUSAP	BC024772	16	38
FANCL	BC054517	16	39
NPM	BC012566	16	40
ZN207	BC008023	16	41
BCL10	BC053617	16	43
ZNF34	BC004480	16	45
PUF60	BC011265	16	46
ZN410	BC050683	16	47
TRIB3	BC019363	16	48
SYTL2	BC015540	16	49
LARP6	BC006082	16	50
NAA20	BC005181	16	51

TBRG1	BC109269	16	52
PARP3	BC014260	16	53
PSMD5	BC014478	16	54
SIR6	BC004218	16	55
CN37	BC011046	16	57
PPIL4	BC020986	16	58
AN32A	BC125143	16	60
OPTN	BC032762	16	61
EIF3L	BC001101	16	62
DSN1	BC058899	16	63
BHE40	BC082238	16	64
I20L2	BC000575	17	1
ARRB1	BC003636	17	2
PI3R5	BC028212	17	3
NEP1	BC055314	17	4
UBP48	BC011576	17	5
CENPR	BC009929	17	6
SYRC	BC000528	17	7
HEY2	BC007707	17	9
NR1H2	BC007790	17	11
LS14B	BC054888	17	12
CBS	BC000440	17	15
CRBA1	BC069537	17	16
Z385A	BC029752	17	17
PRKN2	BC022014	17	18
HMGN1	BC000075	17	19
PRI2	BC017833	17	20
FAM9C	BC127957	17	21
RIR2	BC001886	17	22
EPN1	BC044651	17	23
ELL3	BC006548	17	24
TFAP4	BC010576	17	25
IFRD1	BC001272	17	26
AKTS1	BC015562	17	27
H2BFS	BC126339	17	28
LMBL4	BC039316	17	29
GMCLL	BC024184	17	30
SUPT3	BC050384	17	31
SBDS	BC065700	17	32
WDR74	BC006351	17	33
DNJC7	BC033772	17	34
EXOS1	BC022067	17	35
H2B2E	BC096121	17	36
ADIP	BC033637	17	37
STIP1	BC002987	17	38
HDAC8	BC050433	17	39

CCNG1	BC007093	17	40
ACTB	BC001301	17	41
SP100	BC011562	17	42
RAB8A	BC002977	17	43
STP2	BC096135	17	44
ZN765	BC017357	17	46
BYST	BC050645	17	47
KPCB	BC036472	17	48
RNF8	BC007517	17	50
SH3L3	BC030135	17	51
DUS21	BC119755	17	52
CNDH2	BC009441	17	53
TBX22	BC014194	17	54
NLTP	BC067108	17	55
RIPL1	BC080626	17	56
DRG1	BC019285	17	57
ANXA7	BC002632	17	58
SPC25	BC022255	17	59
RN151	BC113014	17	60
ZN205	BC002810	17	61
NUP85	BC000697	17	62
B2L13	BC003032	17	63
HMBX1	BC069242	17	64
I20L2	BC000575	18	1
ARRB1	BC003636	18	2
PI3R5	BC028212	18	3
NEP1	BC055314	18	4
UBP48	BC011576	18	5
CENPR	BC009929	18	6
SYRC	BC000528	18	7
HEY2	BC007707	18	9
NR1H2	BC007790	18	11
LS14B	BC054888	18	12
CBS	BC000440	18	15
CRBA1	BC069537	18	16
Z385A	BC029752	18	17
PRKN2	BC022014	18	18
HMGN1	BC000075	18	19
PRI2	BC017833	18	20
FAM9C	BC127957	18	21
RIR2	BC001886	18	22
EPN1	BC044651	18	23
ELL3	BC006548	18	24
TFAP4	BC010576	18	25
IFRD1	BC001272	18	26
AKTS1	BC015562	18	27

H2BFS	BC126339	18	28
LMBL4	BC039316	18	29
GMCLL	BC024184	18	30
SUPT3	BC050384	18	31
SBDS	BC065700	18	32
WDR74	BC006351	18	33
DNJC7	BC033772	18	34
EXOS1	BC022067	18	35
H2B2E	BC096121	18	36
ADIP	BC033637	18	37
STIP1	BC002987	18	38
HDAC8	BC050433	18	39
CCNG1	BC007093	18	40
ACTB	BC001301	18	41
SP100	BC011562	18	42
RAB8A	BC002977	18	43
STP2	BC096135	18	44
ZN765	BC017357	18	46
BYST	BC050645	18	47
KPCB	BC036472	18	48
RNF8	BC007517	18	50
SH3L3	BC030135	18	51
DUS21	BC119755	18	52
CNDH2	BC009441	18	53
TBX22	BC014194	18	54
NLTP	BC067108	18	55
RIPL1	BC080626	18	56
DRG1	BC019285	18	57
ANXA7	BC002632	18	58
SPC25	BC022255	18	59
RN151	BC113014	18	60
ZN205	BC002810	18	61
NUP85	BC000697	18	62
B2L13	BC003032	18	63
HMBX1	BC069242	18	64
I20L2	BC000575	19	1
ARRB1	BC003636	19	2
PI3R5	BC028212	19	3
NEP1	BC055314	19	4
UBP48	BC011576	19	5
CENPR	BC009929	19	6
SYRC	BC000528	19	7
HEY2	BC007707	19	9
NR1H2	BC007790	19	11
LS14B	BC054888	19	12
CBS	BC000440	19	15

CRBA1	BC069537	19	16
Z385A	BC029752	19	17
PRKN2	BC022014	19	18
HMGN1	BC000075	19	19
PRI2	BC017833	19	20
FAM9C	BC127957	19	21
RIR2	BC001886	19	22
EPN1	BC044651	19	23
ELL3	BC006548	19	24
TFAP4	BC010576	19	25
IFRD1	BC001272	19	26
AKTS1	BC015562	19	27
H2BFS	BC126339	19	28
LMBL4	BC039316	19	29
GMCLL	BC024184	19	30
SUPT3	BC050384	19	31
SBDS	BC065700	19	32
WDR74	BC006351	19	33
DNJC7	BC033772	19	34
EXOS1	BC022067	19	35
H2B2E	BC096121	19	36
ADIP	BC033637	19	37
STIP1	BC002987	19	38
HDAC8	BC050433	19	39
CCNG1	BC007093	19	40
ACTB	BC001301	19	41
SP100	BC011562	19	42
RAB8A	BC002977	19	43
STP2	BC096135	19	44
ZN765	BC017357	19	46
BYST	BC050645	19	47
KPCB	BC036472	19	48
RNF8	BC007517	19	50
SH3L3	BC030135	19	51
DUS21	BC119755	19	52
CNDH2	BC009441	19	53
TBX22	BC014194	19	54
NLTP	BC067108	19	55
RIPL1	BC080626	19	56
DRG1	BC019285	19	57
ANXA7	BC002632	19	58
SPC25	BC022255	19	59
RN151	BC113014	19	60
ZN205	BC002810	19	61
NUP85	BC000697	19	62
B2L13	BC003032	19	63

HMBX1	BC069242	19	64
I20L2	BC000575	20	1
ARRB1	BC003636	20	2
PI3R5	BC028212	20	3
NEP1	BC055314	20	4
UBP48	BC011576	20	5
CENPR	BC009929	20	6
SYRC	BC000528	20	7
HEY2	BC007707	20	9
NR1H2	BC007790	20	11
LS14B	BC054888	20	12
CBS	BC000440	20	15
CRBA1	BC069537	20	16
Z385A	BC029752	20	17
PRKN2	BC022014	20	18
HMGN1	BC000075	20	19
PRI2	BC017833	20	20
FAM9C	BC127957	20	21
RIR2	BC001886	20	22
EPN1	BC044651	20	23
ELL3	BC006548	20	24
TFAP4	BC010576	20	25
IFRD1	BC001272	20	26
AKTS1	BC015562	20	27
H2BFS	BC126339	20	28
LMBL4	BC039316	20	29
GMCLL	BC024184	20	30
SUPT3	BC050384	20	31
SBDS	BC065700	20	32
WDR74	BC006351	20	33
DNJC7	BC033772	20	34
EXOS1	BC022067	20	35
H2B2E	BC096121	20	36
ADIP	BC033637	20	37
STIP1	BC002987	20	38
HDAC8	BC050433	20	39
CCNG1	BC007093	20	40
ACTB	BC001301	20	41
SP100	BC011562	20	42
RAB8A	BC002977	20	43
STP2	BC096135	20	44
ZN765	BC017357	20	46
BYST	BC050645	20	47
KPCB	BC036472	20	48
RNF8	BC007517	20	50
SH3L3	BC030135	20	51

DUS21	BC119755	20	52
CNDH2	BC009441	20	53
TBX22	BC014194	20	54
NLTP	BC067108	20	55
RIPL1	BC080626	20	56
DRG1	BC019285	20	57
ANXA7	BC002632	20	58
SPC25	BC022255	20	59
RN151	BC113014	20	60
ZN205	BC002810	20	61
NUP85	BC000697	20	62
B2L13	BC003032	20	63
HMBX1	BC069242	20	64
FOS	BC004490	21	2
SPS2L	BC018736	21	3
TGIF2	BC006549	21	4
TLE6	BC020206	21	5
SSX3	BC005904	21	6
RARB	BC060794	21	7
CCND1	BC023620	21	8
ICA69	BC005922	21	9
PPIP1	BC008602	21	10
STK3	BC010640	21	11
SPNXC	BC054023	21	12
CHMP7	BC019110	21	13
ZSA5A	BC002636	21	14
ZC12A	BC005001	21	15
H1T	BC069517	21	16
TRI69	BC033314	21	17
HCFC2	BC006558	21	18
ID3	BC003107	21	19
EMSY	BC029375	21	20
PDS5B	BC039256	21	21
SHLB2	BC014635	21	22
PRC1	BC005140	21	23
ZNF3	BC025265	21	24
AIM2	BC010940	21	25
MP2K5	BC008838	21	26
RBTN2	BC034041	21	27
P121C	BC130587	21	28
FBW1A	BC027994	21	29
DYRK2	BC005809	21	30
MIPO1	BC035870	21	31
BRMS1	BC009834	21	32
EFC4B	BC004524	21	33
RCAN1	BC002864	21	35

H2B1C	BC096120	21	36
HNRLL	BC017480	21	37
ERF	BC022231	21	38
FOXR2	BC012934	21	39
HNRPC	BC008423	21	40
SEPT2	BC033559	21	41
NUSAP	BC001308	21	42
RGS20	BC063490	21	43
H31	BC127610	21	44
WDR62	BC017261	21	45
TOE1	BC009364	21	46
CELF6	BC033838	21	47
ABC3H	BC069023	21	48
MTERF	BC000965	21	49
ZN212	BC022785	21	50
SLIRP	BC017895	21	51
NXT2	BC120984	21	52
GLYM	BC013677	21	54
PSB10	BC052369	21	55
MNDA	BC032319	21	57
K2C7	BC002700	21	58
RLP24	BC028672	21	59
ZN701	BC130351	21	60
COIL	BC010385	21	61
RBM5	BC002957	21	62
NUP43	BC065028	21	63
RBY1A	BC070298	21	64
FOS	BC004490	22	2
SPS2L	BC018736	22	3
TGIF2	BC006549	22	4
TLE6	BC020206	22	5
SSX3	BC005904	22	6
RARB	BC060794	22	7
CCND1	BC023620	22	8
ICA69	BC005922	22	9
PPIP1	BC008602	22	10
STK3	BC010640	22	11
SPNXC	BC054023	22	12
CHMP7	BC019110	22	13
ZSA5A	BC002636	22	14
ZC12A	BC005001	22	15
H1T	BC069517	22	16
TRI69	BC033314	22	17
HCFC2	BC006558	22	18
ID3	BC003107	22	19
EMSY	BC029375	22	20

PDS5B	BC039256	22	21
SHLB2	BC014635	22	22
PRC1	BC005140	22	23
ZNF3	BC025265	22	24
AIM2	BC010940	22	25
MP2K5	BC008838	22	26
RBTN2	BC034041	22	27
P121C	BC130587	22	28
FBW1A	BC027994	22	29
DYRK2	BC005809	22	30
MIPO1	BC035870	22	31
BRMS1	BC009834	22	32
EFC4B	BC004524	22	33
RCAN1	BC002864	22	35
H2B1C	BC096120	22	36
HNRLL	BC017480	22	37
ERF	BC022231	22	38
FOXR2	BC012934	22	39
HNRPC	BC008423	22	40
SEPT2	BC033559	22	41
NUSAP	BC001308	22	42
RGS20	BC063490	22	43
H31	BC127610	22	44
WDR62	BC017261	22	45
TOE1	BC009364	22	46
CELF6	BC033838	22	47
ABC3H	BC069023	22	48
MTERF	BC000965	22	49
ZN212	BC022785	22	50
SLIRP	BC017895	22	51
NXT2	BC120984	22	52
GLYM	BC013677	22	54
PSB10	BC052369	22	55
MNDA	BC032319	22	57
K2C7	BC002700	22	58
RLP24	BC028672	22	59
ZN701	BC130351	22	60
COIL	BC010385	22	61
RBM5	BC002957	22	62
NUP43	BC065028	22	63
RBY1A	BC070298	22	64
FOS	BC004490	23	2
SPS2L	BC018736	23	3
TGIF2	BC006549	23	4
TLE6	BC020206	23	5
SSX3	BC005904	23	6

RARB	BC060794	23	7
CCND1	BC023620	23	8
ICA69	BC005922	23	9
PPIP1	BC008602	23	10
STK3	BC010640	23	11
SPNXC	BC054023	23	12
CHMP7	BC019110	23	13
ZSA5A	BC002636	23	14
ZC12A	BC005001	23	15
H1T	BC069517	23	16
TRI69	BC033314	23	17
HCFC2	BC006558	23	18
ID3	BC003107	23	19
EMSY	BC029375	23	20
PDS5B	BC039256	23	21
SHLB2	BC014635	23	22
PRC1	BC005140	23	23
ZNF3	BC025265	23	24
AIM2	BC010940	23	25
MP2K5	BC008838	23	26
RBTN2	BC034041	23	27
P121C	BC130587	23	28
FBW1A	BC027994	23	29
DYRK2	BC005809	23	30
MIPO1	BC035870	23	31
BRMS1	BC009834	23	32
EFC4B	BC004524	23	33
RCAN1	BC002864	23	35
H2B1C	BC096120	23	36
HNRLL	BC017480	23	37
ERF	BC022231	23	38
FOXR2	BC012934	23	39
HNRPC	BC008423	23	40
SEPT2	BC033559	23	41
NUSAP	BC001308	23	42
RGS20	BC063490	23	43
H31	BC127610	23	44
WDR62	BC017261	23	45
TOE1	BC009364	23	46
CELF6	BC033838	23	47
ABC3H	BC069023	23	48
MTERF	BC000965	23	49
ZN212	BC022785	23	50
SLIRP	BC017895	23	51
NXT2	BC120984	23	52
GLYM	BC013677	23	54

PSB10	BC052369	23	55
MNDA	BC032319	23	57
K2C7	BC002700	23	58
RLP24	BC028672	23	59
ZN701	BC130351	23	60
COIL	BC010385	23	61
RBM5	BC002957	23	62
NUP43	BC065028	23	63
RBY1A	BC070298	23	64
FOS	BC004490	24	2
SPS2L	BC018736	24	3
TGIF2	BC006549	24	4
TLE6	BC020206	24	5
SSX3	BC005904	24	6
RARB	BC060794	24	7
CCND1	BC023620	24	8
ICA69	BC005922	24	9
PPIP1	BC008602	24	10
STK3	BC010640	24	11
SPNXC	BC054023	24	12
CHMP7	BC019110	24	13
ZSA5A	BC002636	24	14
ZC12A	BC005001	24	15
H1T	BC069517	24	16
TRI69	BC033314	24	17
HCFC2	BC006558	24	18
ID3	BC003107	24	19
EMSY	BC029375	24	20
PDS5B	BC039256	24	21
SHLB2	BC014635	24	22
PRC1	BC005140	24	23
ZNF3	BC025265	24	24
AIM2	BC010940	24	25
MP2K5	BC008838	24	26
RBTN2	BC034041	24	27
P121C	BC130587	24	28
FBW1A	BC027994	24	29
DYRK2	BC005809	24	30
MIPO1	BC035870	24	31
BRMS1	BC009834	24	32
EFC4B	BC004524	24	33
RCAN1	BC002864	24	35
H2B1C	BC096120	24	36
HNRLL	BC017480	24	37
ERF	BC022231	24	38
FOXR2	BC012934	24	39

HNRPC	BC008423	24	40
SEPT2	BC033559	24	41
NUSAP	BC001308	24	42
RGS20	BC063490	24	43
H31	BC127610	24	44
WDR62	BC017261	24	45
TOE1	BC009364	24	46
CELF6	BC033838	24	47
ABC3H	BC069023	24	48
MTERF	BC000965	24	49
ZN212	BC022785	24	50
SLIRP	BC017895	24	51
NXT2	BC120984	24	52
GLYM	BC013677	24	54
PSB10	BC052369	24	55
MNDA	BC032319	24	57
K2C7	BC002700	24	58
RLP24	BC028672	24	59
ZN701	BC130351	24	60
COIL	BC010385	24	61
RBM5	BC002957	24	62
NUP43	BC065028	24	63
RBY1A	BC070298	24	64
SSH3	BC004176	25	1
ZPR1	BC004256	25	3
CLIC2	BC022305	25	4
KRR1	BC016778	25	5
FANK1	BC024189	25	6
C43BP	BC000102	25	7
CRYAA	BC069528	25	8
ASNA	BC002651	25	9
RPA49	BC001337	25	10
RBY1F	BC030018	25	11
H4	BC054014	25	12
HNRPF	BC016736	25	13
K1967	BC018269	25	14
MEN1	BC002544	25	15
HXA6	BC069497	25	16
PCGF2	BC004858	25	17
DUS6	BC003143	25	18
ZN695	BC041082	25	20
GIT2	BC014223	25	21
PO2F2	BC006101	25	22
ZN133	BC001887	25	23
UCKL1	BC033078	25	24
PSD13	BC001100	25	25

PPAC	BC007422	25	27
SP110	BC019059	25	29
ZN680	BC030700	25	30
THOC6	BC050674	25	31
RAD9B	BC068031	25	32
CABL1	BC037218	25	33
ECHB	BC014572	25	34
SALL2	BC024245	25	35
BEX1	BC126427	25	36
NR4A2	BC009288	25	37
SRP54	BC003389	25	38
MBNL1	BC043493	25	39
SOX2	BC013923	25	40
DEK	BC035259	25	41
NEK11	BC028587	25	42
RPAB1	BC004441	25	43
DAOA	BC121091	25	44
ZBT7B	BC012070	25	45
MT1X	BC032338	25	46
BAG5	BC050551	25	47
MK11	BC027933	25	49
NFIL3	BC008197	25	50
ZCHC7	BC022434	25	51
ZN137	BC110455	25	52
DCP1A	BC007439	25	53
GMCL1	BC007420	25	54
XRCC2	BC042137	25	55
TRBP2	BC005860	25	57
PUF60	BC009734	25	58
RBM4	BC021120	25	59
CHTOP	BC120961	25	60
EAF1	BC041329	25	61
EP400	BC066974	25	62
USF2	BC049821	25	63
OGFR	BC014137	25	64
SSH3	BC004176	26	1
ZPR1	BC004256	26	3
CLIC2	BC022305	26	4
KRR1	BC016778	26	5
FANK1	BC024189	26	6
C43BP	BC000102	26	7
CRYAA	BC069528	26	8
ASNA	BC002651	26	9
RPA49	BC001337	26	10
RBY1F	BC030018	26	11
H4	BC054014	26	12

HNRPF	BC016736	26	13
K1967	BC018269	26	14
MEN1	BC002544	26	15
HXA6	BC069497	26	16
PCGF2	BC004858	26	17
DUS6	BC003143	26	18
ZN695	BC041082	26	20
GIT2	BC014223	26	21
PO2F2	BC006101	26	22
ZN133	BC001887	26	23
UCKL1	BC033078	26	24
PSD13	BC001100	26	25
PPAC	BC007422	26	27
SP110	BC019059	26	29
ZN680	BC030700	26	30
THOC6	BC050674	26	31
RAD9B	BC068031	26	32
CABL1	BC037218	26	33
ECHB	BC014572	26	34
SALL2	BC024245	26	35
BEX1	BC126427	26	36
NR4A2	BC009288	26	37
SRP54	BC003389	26	38
MBNL1	BC043493	26	39
SOX2	BC013923	26	40
DEK	BC035259	26	41
NEK11	BC028587	26	42
RPAB1	BC004441	26	43
DAOA	BC121091	26	44
ZBT7B	BC012070	26	45
MT1X	BC032338	26	46
BAG5	BC050551	26	47
MK11	BC027933	26	49
NFIL3	BC008197	26	50
ZCHC7	BC022434	26	51
ZN137	BC110455	26	52
DCP1A	BC007439	26	53
GMCL1	BC007420	26	54
XRCC2	BC042137	26	55
TRBP2	BC005860	26	57
PUF60	BC009734	26	58
RBM4	BC021120	26	59
CHTOP	BC120961	26	60
EAF1	BC041329	26	61
EP400	BC066974	26	62
USF2	BC049821	26	63

OGFR	BC014137	26	64
SSH3	BC004176	27	1
ZPR1	BC004256	27	3
CLIC2	BC022305	27	4
KRR1	BC016778	27	5
FANK1	BC024189	27	6
C43BP	BC000102	27	7
CRYAA	BC069528	27	8
ASNA	BC002651	27	9
RPA49	BC001337	27	10
RBY1F	BC030018	27	11
H4	BC054014	27	12
HNRPF	BC016736	27	13
K1967	BC018269	27	14
MEN1	BC002544	27	15
HXA6	BC069497	27	16
PCGF2	BC004858	27	17
DUS6	BC003143	27	18
ZN695	BC041082	27	20
GIT2	BC014223	27	21
PO2F2	BC006101	27	22
ZN133	BC001887	27	23
UCKL1	BC033078	27	24
PSD13	BC001100	27	25
PPAC	BC007422	27	27
SP110	BC019059	27	29
ZN680	BC030700	27	30
THOC6	BC050674	27	31
RAD9B	BC068031	27	32
CABL1	BC037218	27	33
ECHB	BC014572	27	34
SALL2	BC024245	27	35
BEX1	BC126427	27	36
NR4A2	BC009288	27	37
SRP54	BC003389	27	38
MBNL1	BC043493	27	39
SOX2	BC013923	27	40
DEK	BC035259	27	41
NEK11	BC028587	27	42
RPAB1	BC004441	27	43
DAOA	BC121091	27	44
ZBT7B	BC012070	27	45
MT1X	BC032338	27	46
BAG5	BC050551	27	47
MK11	BC027933	27	49
NFIL3	BC008197	27	50

ZCHC7	BC022434	27	51
ZN137	BC110455	27	52
DCP1A	BC007439	27	53
GMCL1	BC007420	27	54
XRCC2	BC042137	27	55
TRBP2	BC005860	27	57
PUF60	BC009734	27	58
RBM4	BC021120	27	59
CHTOP	BC120961	27	60
EAF1	BC041329	27	61
EP400	BC066974	27	62
USF2	BC049821	27	63
OGFR	BC014137	27	64
SSH3	BC004176	28	1
ZPR1	BC004256	28	3
CLIC2	BC022305	28	4
KRR1	BC016778	28	5
FANK1	BC024189	28	6
C43BP	BC000102	28	7
CRYAA	BC069528	28	8
ASNA	BC002651	28	9
RPA49	BC001337	28	10
RBY1F	BC030018	28	11
H4	BC054014	28	12
HNRPF	BC016736	28	13
K1967	BC018269	28	14
MEN1	BC002544	28	15
HXA6	BC069497	28	16
PCGF2	BC004858	28	17
DUS6	BC003143	28	18
ZN695	BC041082	28	20
GIT2	BC014223	28	21
PO2F2	BC006101	28	22
ZN133	BC001887	28	23
UCKL1	BC033078	28	24
PSD13	BC001100	28	25
PPAC	BC007422	28	27
SP110	BC019059	28	29
ZN680	BC030700	28	30
THOC6	BC050674	28	31
RAD9B	BC068031	28	32
CABL1	BC037218	28	33
ECHB	BC014572	28	34
SALL2	BC024245	28	35
BEX1	BC126427	28	36
NR4A2	BC009288	28	37

SRP54	BC003389	28	38
MBNL1	BC043493	28	39
SOX2	BC013923	28	40
DEK	BC035259	28	41
NEK11	BC028587	28	42
RPAB1	BC004441	28	43
DAOA	BC121091	28	44
ZBT7B	BC012070	28	45
MT1X	BC032338	28	46
BAG5	BC050551	28	47
MK11	BC027933	28	49
NFIL3	BC008197	28	50
ZCHC7	BC022434	28	51
ZN137	BC110455	28	52
DCP1A	BC007439	28	53
GMCL1	BC007420	28	54
XRCC2	BC042137	28	55
TRBP2	BC005860	28	57
PUF60	BC009734	28	58
RBM4	BC021120	28	59
CHTOP	BC120961	28	60
EAF1	BC041329	28	61
EP400	BC066974	28	62
USF2	BC049821	28	63
OGFR	BC014137	28	64
CCD86	BC001378	29	1
ZN764	BC008821	29	2
SRSF4	BC002781	29	3
RS6	BC027620	29	4
TPPP	BC131506	29	5
TRIP6	BC002680	29	6
NUD12	BC041099	29	7
CABP1	BC015006	29	8
LDB1	BC000482	29	9
ZN679	BC033523	29	10
MED27	BC002878	29	11
F188A	BC020605	29	12
ARL4C	BC089043	29	13
H2AY	BC013331	29	14
PIAS4	BC066895	29	15
F110B	BC024294	29	16
RCC1	BC007300	29	18
RPC10	BC011932	29	19
VP37A	BC022363	29	20
LKHA4	BC032528	29	21
GRHL3	BC036890	29	23

TNR6A	BC068209	29	24
QKI	BC019917	29	25
CRBN	BC017419	29	26
FABP7	BC012299	29	27
VP33A	BC016617	29	29
MUTYH	BC003178	29	30
OVOL1	BC059408	29	31
CENPP	BC071726	29	32
MCM9	BC031658	29	33
NOL6	BC008298	29	35
H2B3B	BC100855	29	36
ELF2	BC034951	29	37
NIPA	BC011551	29	38
RAB3I	BC059358	29	39
SPOPL	BC071613	29	40
GABP2	BC027033	29	42
CR3L2	BC063666	29	43
SSX4	BC103864	29	44
RFX5	BC017471	29	45
AAKB2	BC053610	29	47
PSME4	BC071768	29	48
ACTT3	BC007289	29	49
NUP54	BC012559	29	50
SPRY4	BC020844	29	51
FBX32	BC120963	29	52
GNL3L	BC011720	29	53
AAAS	BC000659	29	54
MAT2B	BC066645	29	55
MYBB	BC007585	29	56
RL3	BC006483	29	57
CUL1	BC034318	29	58
DUS22	BC022847	29	59
RAB6C	BC120999	29	60
FOXN2	BC063305	29	62
SSBP3	BC066365	29	63
EED	BC068995	29	64
CCD86	BC001378	30	1
ZN764	BC008821	30	2
SRSF4	BC002781	30	3
RS6	BC027620	30	4
TPPP	BC131506	30	5
TRIP6	BC002680	30	6
NUD12	BC041099	30	7
CABP1	BC015006	30	8
LDB1	BC000482	30	9
ZN679	BC033523	30	10

MED27	BC002878	30	11
F188A	BC020605	30	12
ARL4C	BC089043	30	13
H2AY	BC013331	30	14
PIAS4	BC066895	30	15
F110B	BC024294	30	16
RCC1	BC007300	30	18
RPC10	BC011932	30	19
VP37A	BC022363	30	20
LKHA4	BC032528	30	21
GRHL3	BC036890	30	23
TNR6A	BC068209	30	24
QKI	BC019917	30	25
CRBN	BC017419	30	26
FABP7	BC012299	30	27
VP33A	BC016617	30	29
MUTYH	BC003178	30	30
OVOL1	BC059408	30	31
CENPP	BC071726	30	32
MCM9	BC031658	30	33
NOL6	BC008298	30	35
H2B3B	BC100855	30	36
ELF2	BC034951	30	37
NIPA	BC011551	30	38
RAB3I	BC059358	30	39
SPOPL	BC071613	30	40
GABP2	BC027033	30	42
CR3L2	BC063666	30	43
SSX4	BC103864	30	44
RFX5	BC017471	30	45
AAKB2	BC053610	30	47
PSME4	BC071768	30	48
ACTT3	BC007289	30	49
NUP54	BC012559	30	50
SPRY4	BC020844	30	51
FBX32	BC120963	30	52
GNL3L	BC011720	30	53
AAAS	BC000659	30	54
MAT2B	BC066645	30	55
MYBB	BC007585	30	56
RL3	BC006483	30	57
CUL1	BC034318	30	58
DUS22	BC022847	30	59
RAB6C	BC120999	30	60
FOXN2	BC063305	30	62
SSBP3	BC066365	30	63

EED	BC068995	30	64
CCD86	BC001378	31	1
ZN764	BC008821	31	2
SRSF4	BC002781	31	3
RS6	BC027620	31	4
TPPP	BC131506	31	5
TRIP6	BC002680	31	6
NUD12	BC041099	31	7
CABP1	BC015006	31	8
LDB1	BC000482	31	9
ZN679	BC033523	31	10
MED27	BC002878	31	11
F188A	BC020605	31	12
ARL4C	BC089043	31	13
H2AY	BC013331	31	14
PIAS4	BC066895	31	15
F110B	BC024294	31	16
RCC1	BC007300	31	18
RPC10	BC011932	31	19
VP37A	BC022363	31	20
LKHA4	BC032528	31	21
GRHL3	BC036890	31	23
TNR6A	BC068209	31	24
QKI	BC019917	31	25
CRBN	BC017419	31	26
FABP7	BC012299	31	27
VP33A	BC016617	31	29
MUTYH	BC003178	31	30
OVOL1	BC059408	31	31
CENPP	BC071726	31	32
MCM9	BC031658	31	33
NOL6	BC008298	31	35
H2B3B	BC100855	31	36
ELF2	BC034951	31	37
NIPA	BC011551	31	38
RAB3I	BC059358	31	39
SPOPL	BC071613	31	40
GABP2	BC027033	31	42
CR3L2	BC063666	31	43
SSX4	BC103864	31	44
RFX5	BC017471	31	45
AAKB2	BC053610	31	47
PSME4	BC071768	31	48
ACTT3	BC007289	31	49
NUP54	BC012559	31	50
SPRY4	BC020844	31	51

FBX32	BC120963	31	52
GNL3L	BC011720	31	53
AAAS	BC000659	31	54
MAT2B	BC066645	31	55
MYBB	BC007585	31	56
RL3	BC006483	31	57
CUL1	BC034318	31	58
DUS22	BC022847	31	59
RAB6C	BC120999	31	60
FOXN2	BC063305	31	62
SSBP3	BC066365	31	63
EED	BC068995	31	64
CCD86	BC001378	32	1
ZN764	BC008821	32	2
SRSF4	BC002781	32	3
RS6	BC027620	32	4
TPPP	BC131506	32	5
TRIP6	BC002680	32	6
NUD12	BC041099	32	7
CABP1	BC015006	32	8
LDB1	BC000482	32	9
ZN679	BC033523	32	10
MED27	BC002878	32	11
F188A	BC020605	32	12
ARL4C	BC089043	32	13
H2AY	BC013331	32	14
PIAS4	BC066895	32	15
F110B	BC024294	32	16
RCC1	BC007300	32	18
RPC10	BC011932	32	19
VP37A	BC022363	32	20
LKHA4	BC032528	32	21
GRHL3	BC036890	32	23
TNR6A	BC068209	32	24
QKI	BC019917	32	25
CRBN	BC017419	32	26
FABP7	BC012299	32	27
VP33A	BC016617	32	29
MUTYH	BC003178	32	30
OVOL1	BC059408	32	31
CENPP	BC071726	32	32
MCM9	BC031658	32	33
NOL6	BC008298	32	35
H2B3B	BC100855	32	36
ELF2	BC034951	32	37
NIPA	BC011551	32	38

RAB3I	BC059358	32	39
SPOPL	BC071613	32	40
GABP2	BC027033	32	42
CR3L2	BC063666	32	43
SSX4	BC103864	32	44
RFX5	BC017471	32	45
AAKB2	BC053610	32	47
PSME4	BC071768	32	48
ACTT3	BC007289	32	49
NUP54	BC012559	32	50
SPRY4	BC020844	32	51
FBX32	BC120963	32	52
GNL3L	BC011720	32	53
AAAS	BC000659	32	54
MAT2B	BC066645	32	55
MYBB	BC007585	32	56
RL3	BC006483	32	57
CUL1	BC034318	32	58
DUS22	BC022847	32	59
RAB6C	BC120999	32	60
FOXN2	BC063305	32	62
SSBP3	BC066365	32	63
EED	BC068995	32	64
DND1	BC033496	33	1
ZN550	BC034810	33	2
RGS7	BC022009	33	3
HM20B	BC021585	33	4
PIN4	BC112281	33	5
PTN23	BC004881	33	6
ERR3	BC064700	33	7
PNMA2	BC062301	33	8
BDH	BC011964	33	9
ZNF25	BC036038	33	10
STF1	BC032501	33	11
PSA6	BC023659	33	12
NFIC	BC012120	33	13
GBB5	BC013997	33	14
FANCG	BC000032	33	15
ZN187	BC013951	33	16
EWS	BC000527	33	17
NCK1	BC006403	33	18
TRIPB	BC002656	33	19
PPL13	BC022257	33	20
LPXN	BC019035	33	21
TFCP2	BC003634	33	22
TRI27	BC066924	33	23

KLF9	BC069431	33	24
JUNB	BC004250	33	25
VMA5A	BC001234	33	26
MPLKI	BC026265	33	28
PDE9A	BC009047	33	29
AATF	BC000591	33	31
PRUNE	BC063481	33	32
ISL1	BC031213	33	33
ZMIZ2	BC021924	33	34
NCS1	BC004856	33	35
T53G3	BC096733	33	36
ACK1	BC028164	33	37
ZN643	BC017498	33	38
SKAP1	BC047870	33	39
CSN7A	BC011789	33	40
RD23A	BC014026	33	41
ZBT26	BC018748	33	42
PMF1	BC065031	33	43
IF2B2	BC021290	33	45
CR3L1	BC014097	33	46
GABP1	BC050702	33	47
VP37A	BC067754	33	48
ZN343	BC011862	33	49
PUS3	BC004822	33	50
ORC6	BC063565	33	51
DPPA3	BC125145	33	52
UBQL1	BC039294	33	53
ZBT45	BC024738	33	54
MO4L2	BC056899	33	55
SIR7	BC017305	33	57
DCTN4	BC026323	33	58
MOFA1	BC022797	33	59
RPA12	BC051741	33	60
XRN2	BC006417	33	61
CCD89	BC044814	33	62
FBX25	BC020249	33	63
FL2D	BC069192	33	64
DND1	BC033496	34	1
ZN550	BC034810	34	2
RGS7	BC022009	34	3
HM20B	BC021585	34	4
PIN4	BC112281	34	5
PTN23	BC004881	34	6
ERR3	BC064700	34	7
PNMA2	BC062301	34	8
BDH	BC011964	34	9

ZNF25	BC036038	34	10
STF1	BC032501	34	11
PSA6	BC023659	34	12
NFIC	BC012120	34	13
GBB5	BC013997	34	14
FANCG	BC000032	34	15
ZN187	BC013951	34	16
EWS	BC000527	34	17
NCK1	BC006403	34	18
TRIPB	BC002656	34	19
PPL13	BC022257	34	20
LPXN	BC019035	34	21
TFCP2	BC003634	34	22
TRI27	BC066924	34	23
KLF9	BC069431	34	24
JUNB	BC004250	34	25
VMA5A	BC001234	34	26
MPLKI	BC026265	34	28
PDE9A	BC009047	34	29
AATF	BC000591	34	31
PRUNE	BC063481	34	32
ISL1	BC031213	34	33
ZMIZ2	BC021924	34	34
NCS1	BC004856	34	35
T53G3	BC096733	34	36
ACK1	BC028164	34	37
ZN643	BC017498	34	38
SKAP1	BC047870	34	39
CSN7A	BC011789	34	40
RD23A	BC014026	34	41
ZBT26	BC018748	34	42
PMF1	BC065031	34	43
IF2B2	BC021290	34	45
CR3L1	BC014097	34	46
GABP1	BC050702	34	47
VP37A	BC067754	34	48
ZN343	BC011862	34	49
PUS3	BC004822	34	50
ORC6	BC063565	34	51
DPPA3	BC125145	34	52
UBQL1	BC039294	34	53
ZBT45	BC024738	34	54
MO4L2	BC056899	34	55
SIR7	BC017305	34	57
DCTN4	BC026323	34	58
MOFA1	BC022797	34	59

RPA12	BC051741	34	60
XRN2	BC006417	34	61
CCD89	BC044814	34	62
FBX25	BC020249	34	63
FL2D	BC069192	34	64
DND1	BC033496	35	1
ZN550	BC034810	35	2
RGS7	BC022009	35	3
HM20B	BC021585	35	4
PIN4	BC112281	35	5
PTN23	BC004881	35	6
ERR3	BC064700	35	7
PNMA2	BC062301	35	8
BDH	BC011964	35	9
ZNF25	BC036038	35	10
STF1	BC032501	35	11
PSA6	BC023659	35	12
NFIC	BC012120	35	13
GBB5	BC013997	35	14
FANCG	BC000032	35	15
ZN187	BC013951	35	16
EWS	BC000527	35	17
NCK1	BC006403	35	18
TRIPB	BC002656	35	19
PPL13	BC022257	35	20
LPXN	BC019035	35	21
TFCP2	BC003634	35	22
TRI27	BC066924	35	23
KLF9	BC069431	35	24
JUNB	BC004250	35	25
VMA5A	BC001234	35	26
MPLKI	BC026265	35	28
PDE9A	BC009047	35	29
AATF	BC000591	35	31
PRUNE	BC063481	35	32
ISL1	BC031213	35	33
ZMIZ2	BC021924	35	34
NCS1	BC004856	35	35
T53G3	BC096733	35	36
ACK1	BC028164	35	37
ZN643	BC017498	35	38
SKAP1	BC047870	35	39
CSN7A	BC011789	35	40
RD23A	BC014026	35	41
ZBT26	BC018748	35	42
PMF1	BC065031	35	43

IF2B2	BC021290	35	45
CR3L1	BC014097	35	46
GABP1	BC050702	35	47
VP37A	BC067754	35	48
ZN343	BC011862	35	49
PUS3	BC004822	35	50
ORC6	BC063565	35	51
DPPA3	BC125145	35	52
UBQL1	BC039294	35	53
ZBT45	BC024738	35	54
MO4L2	BC056899	35	55
SIR7	BC017305	35	57
DCTN4	BC026323	35	58
MOFA1	BC022797	35	59
RPA12	BC051741	35	60
XRN2	BC006417	35	61
CCD89	BC044814	35	62
FBX25	BC020249	35	63
FL2D	BC069192	35	64
DND1	BC033496	36	1
ZN550	BC034810	36	2
RGS7	BC022009	36	3
HM20B	BC021585	36	4
PIN4	BC112281	36	5
PTN23	BC004881	36	6
ERR3	BC064700	36	7
PNMA2	BC062301	36	8
BDH	BC011964	36	9
ZNF25	BC036038	36	10
STF1	BC032501	36	11
PSA6	BC023659	36	12
NFIC	BC012120	36	13
GBB5	BC013997	36	14
FANCG	BC000032	36	15
ZN187	BC013951	36	16
EWS	BC000527	36	17
NCK1	BC006403	36	18
TRIPB	BC002656	36	19
PPL13	BC022257	36	20
LPXN	BC019035	36	21
TFCP2	BC003634	36	22
TRI27	BC066924	36	23
KLF9	BC069431	36	24
JUNB	BC004250	36	25
VMA5A	BC001234	36	26
MPLKI	BC026265	36	28

PDE9A	BC009047	36	29
AATF	BC000591	36	31
PRUNE	BC063481	36	32
ISL1	BC031213	36	33
ZMIZ2	BC021924	36	34
NCS1	BC004856	36	35
T53G3	BC096733	36	36
ACK1	BC028164	36	37
ZN643	BC017498	36	38
SKAP1	BC047870	36	39
CSN7A	BC011789	36	40
RD23A	BC014026	36	41
ZBT26	BC018748	36	42
PMF1	BC065031	36	43
IF2B2	BC021290	36	45
CR3L1	BC014097	36	46
GABP1	BC050702	36	47
VP37A	BC067754	36	48
ZN343	BC011862	36	49
PUS3	BC004822	36	50
ORC6	BC063565	36	51
DPPA3	BC125145	36	52
UBQL1	BC039294	36	53
ZBT45	BC024738	36	54
MO4L2	BC056899	36	55
SIR7	BC017305	36	57
DCTN4	BC026323	36	58
MOFA1	BC022797	36	59
RPA12	BC051741	36	60
XRN2	BC006417	36	61
CCD89	BC044814	36	62
FBX25	BC020249	36	63
FL2D	BC069192	36	64
TMOD1	BC002660	37	1
KCC1D	BC035745	37	2
KLF4	BC029923	37	3
PSB1	BC020807	37	4
ZN75A	BC119652	37	5
EHF	BC038995	37	6
PREP	BC001150	37	7
ZNF32	BC022842	37	8
RFOX2	BC013115	37	9
KLF15	BC036733	37	10
SMAD1	BC001878	37	11
TWF1	BC022344	37	12
DD19A	BC005162	37	14

CRTC1	BC017075	37	15
KAZRN	BC035501	37	16
PSMD4	BC002365	37	17
RUXE	BC002639	37	19
GALT3	BC056246	37	20
TDT	BC012920	37	22
HIRP3	BC000588	37	23
MEOX1	BC069474	37	24
CDK7	BC000834	37	25
TRI55	BC007750	37	26
DUS18	BC028724	37	27
ZMAT2	BC056668	37	28
TRI41	BC018765	37	29
ARRB2	BC007427	37	30
MAK16	BC050528	37	31
ERCC8	BC009793	37	32
ZNF24	BC003566	37	33
PRUN2	BC022571	37	35
ZN663	BC100775	37	36
KPCI	BC022016	37	37
KPTN	BC009249	37	38
CNOT7	BC060852	37	39
CA114	BC026073	37	41
TAF1A	BC013808	37	42
MYOG	BC053899	37	43
H31	BC096131	37	44
TNIP1	BC012133	37	45
KCC2B	BC019070	37	46
ZN322	BC050425	37	47
FA53A	BC070112	37	48
RING1	BC002922	37	49
CLK3	BC006103	37	50
HXC9	BC053894	37	51
PRDM7	BC107033	37	52
NDC80	BC035617	37	53
MT3	BC013081	37	54
SETBP	BC062338	37	55
DBF4A	BC036045	37	56
RBMS2	BC027863	37	57
AKIP1	BC030996	37	59
INCA1	BC119781	37	60
DTD1	BC045167	37	61
IRF9	BC035716	37	62
DHRS2	BC002786	37	63
HEM1	BC011798	37	64
TMOD1	BC002660	38	1

KCC1D	BC035745	38	2
KLF4	BC029923	38	3
PSB1	BC020807	38	4
ZN75A	BC119652	38	5
EHF	BC038995	38	6
PREP	BC001150	38	7
ZNF32	BC022842	38	8
RFOX2	BC013115	38	9
KLF15	BC036733	38	10
SMAD1	BC001878	38	11
TWF1	BC022344	38	12
DD19A	BC005162	38	14
CRTC1	BC017075	38	15
KAZRN	BC035501	38	16
PSMD4	BC002365	38	17
RUXE	BC002639	38	19
GALT3	BC056246	38	20
TDT	BC012920	38	22
HIRP3	BC000588	38	23
MEOX1	BC069474	38	24
CDK7	BC000834	38	25
TRI55	BC007750	38	26
DUS18	BC028724	38	27
ZMAT2	BC056668	38	28
TRI41	BC018765	38	29
ARRB2	BC007427	38	30
MAK16	BC050528	38	31
ERCC8	BC009793	38	32
ZNF24	BC003566	38	33
PRUN2	BC022571	38	35
ZN663	BC100775	38	36
KPCI	BC022016	38	37
KPTN	BC009249	38	38
CNOT7	BC060852	38	39
CA114	BC026073	38	41
TAF1A	BC013808	38	42
MYOG	BC053899	38	43
H31	BC096131	38	44
TNIP1	BC012133	38	45
KCC2B	BC019070	38	46
ZN322	BC050425	38	47
FA53A	BC070112	38	48
RING1	BC002922	38	49
CLK3	BC006103	38	50
HXC9	BC053894	38	51
PRDM7	BC107033	38	52

NDC80	BC035617	38	53
MT3	BC013081	38	54
SETBP	BC062338	38	55
DBF4A	BC036045	38	56
RBMS2	BC027863	38	57
AKIP1	BC030996	38	59
INCA1	BC119781	38	60
DTD1	BC045167	38	61
IRF9	BC035716	38	62
DHRS2	BC002786	38	63
HEM1	BC011798	38	64
TMOD1	BC002660	39	1
KCC1D	BC035745	39	2
KLF4	BC029923	39	3
PSB1	BC020807	39	4
ZN75A	BC119652	39	5
EHF	BC038995	39	6
PREP	BC001150	39	7
ZNF32	BC022842	39	8
RFOX2	BC013115	39	9
KLF15	BC036733	39	10
SMAD1	BC001878	39	11
TWF1	BC022344	39	12
DD19A	BC005162	39	14
CRTC1	BC017075	39	15
KAZRN	BC035501	39	16
PSMD4	BC002365	39	17
RUXE	BC002639	39	19
GALT3	BC056246	39	20
TDT	BC012920	39	22
HIRP3	BC000588	39	23
MEOX1	BC069474	39	24
CDK7	BC000834	39	25
TRI55	BC007750	39	26
DUS18	BC028724	39	27
ZMAT2	BC056668	39	28
TRI41	BC018765	39	29
ARRB2	BC007427	39	30
MAK16	BC050528	39	31
ERCC8	BC009793	39	32
ZNF24	BC003566	39	33
PRUN2	BC022571	39	35
ZN663	BC100775	39	36
KPCI	BC022016	39	37
KPTN	BC009249	39	38
CNOT7	BC060852	39	39

CA114	BC026073	39	41
TAF1A	BC013808	39	42
MYOG	BC053899	39	43
H31	BC096131	39	44
TNIP1	BC012133	39	45
KCC2B	BC019070	39	46
ZN322	BC050425	39	47
FA53A	BC070112	39	48
RING1	BC002922	39	49
CLK3	BC006103	39	50
HXC9	BC053894	39	51
PRDM7	BC107033	39	52
NDC80	BC035617	39	53
MT3	BC013081	39	54
SETBP	BC062338	39	55
DBF4A	BC036045	39	56
RBMS2	BC027863	39	57
AKIP1	BC030996	39	59
INCA1	BC119781	39	60
DTD1	BC045167	39	61
IRF9	BC035716	39	62
DHRS2	BC002786	39	63
HEM1	BC011798	39	64
TMOD1	BC002660	40	1
KCC1D	BC035745	40	2
KLF4	BC029923	40	3
PSB1	BC020807	40	4
ZN75A	BC119652	40	5
EHF	BC038995	40	6
PREP	BC001150	40	7
ZNF32	BC022842	40	8
RFOX2	BC013115	40	9
KLF15	BC036733	40	10
SMAD1	BC001878	40	11
TWF1	BC022344	40	12
DD19A	BC005162	40	14
CRTC1	BC017075	40	15
KAZRN	BC035501	40	16
PSMD4	BC002365	40	17
RUXE	BC002639	40	19
GALT3	BC056246	40	20
TDT	BC012920	40	22
HIRP3	BC000588	40	23
MEOX1	BC069474	40	24
CDK7	BC000834	40	25
TRI55	BC007750	40	26

DUS18	BC028724	40	27
ZMAT2	BC056668	40	28
TRI41	BC018765	40	29
ARRB2	BC007427	40	30
MAK16	BC050528	40	31
ERCC8	BC009793	40	32
ZNF24	BC003566	40	33
PRUN2	BC022571	40	35
ZN663	BC100775	40	36
KPCI	BC022016	40	37
KPTN	BC009249	40	38
CNOT7	BC060852	40	39
CA114	BC026073	40	41
TAF1A	BC013808	40	42
MYOG	BC053899	40	43
H31	BC096131	40	44
TNIP1	BC012133	40	45
KCC2B	BC019070	40	46
ZN322	BC050425	40	47
FA53A	BC070112	40	48
RING1	BC002922	40	49
CLK3	BC006103	40	50
HXC9	BC053894	40	51
PRDM7	BC107033	40	52
NDC80	BC035617	40	53
MT3	BC013081	40	54
SETBP	BC062338	40	55
DBF4A	BC036045	40	56
RBMS2	BC027863	40	57
AKIP1	BC030996	40	59
INCA1	BC119781	40	60
DTD1	BC045167	40	61
IRF9	BC035716	40	62
DHRS2	BC002786	40	63
HEM1	BC011798	40	64
Z385B	BC038422	41	1
G3BP1	BC006997	41	3
TAP26	BC020647	41	4
WAPL	BC017393	41	5
RGS14	BC014094	41	6
SYK	BC004132	41	7
HEXI2	BC025970	41	8
PCBP2	BC001155	41	9
PEX14	BC006327	41	10
HEXDC	BC035561	41	11
RRP15	BC020641	41	12

NOSTN	BC014189	41	13
HJURP	BC001940	41	14
BCL6B	BC059404	41	15
HESX1	BC069515	41	16
MP2K3	BC032478	41	17
AIPL1	BC012055	41	18
PSDE	BC009524	41	19
ZNF69	BC051309	41	20
TFDP2	BC021113	41	21
TRI27	BC013580	41	22
ZN432	BC002858	41	23
MEIS2	BC001516	41	24
SQSTM	BC001874	41	25
KCC2D	BC032784	41	26
HPGDS	BC020734	41	28
RGAP1	BC032754	41	29
TFDP1	BC011685	41	30
ZRAB2	BC039814	41	31
PAX3	BC063547	41	32
CREB1	BC010636	41	33
GLYC	BC007979	41	34
CDKN3	BC064965	41	35
SH3L2	BC052987	41	36
SP2	BC033814	41	37
IMA1	BC002374	41	38
DMRT1	BC040847	41	39
IMP4	BC010042	41	40
DCC1	BC001531	41	41
CLK1	BC031549	41	42
NTAQ1	BC008781	41	43
H2A2A	BC096705	41	44
IMA7	BC020520	41	45
NMD3	BC013317	41	46
POGZ	BC057773	41	47
FIBP	BC014388	41	48
NFE2	BC005044	41	49
CELF5	BC028101	41	50
STMN2	BC006302	41	51
FAM9B	BC120955	41	52
NXF3	BC031616	41	53
PP2BA	BC025714	41	54
KAT1	BC021262	41	55
NSD3	BC012059	41	56
LANC1	BC028685	41	57
NAB1	BC035724	41	58
IL37	BC020637	41	59

ICLN	BC119634	41	60
ADAD1	BC040229	41	61
PPM1G	BC000057	41	62
LUC7L	BC065198	41	63
Z385B	BC038422	42	1
G3BP1	BC006997	42	3
TAP26	BC020647	42	4
WAPL	BC017393	42	5
RGS14	BC014094	42	6
SYK	BC004132	42	7
HEXI2	BC025970	42	8
PCBP2	BC001155	42	9
PEX14	BC006327	42	10
HEXDC	BC035561	42	11
RRP15	BC020641	42	12
NOSTN	BC014189	42	13
HJURP	BC001940	42	14
BCL6B	BC059404	42	15
HESX1	BC069515	42	16
MP2K3	BC032478	42	17
AIPL1	BC012055	42	18
PSDE	BC009524	42	19
ZNF69	BC051309	42	20
TFDP2	BC021113	42	21
TRI27	BC013580	42	22
ZN432	BC002858	42	23
MEIS2	BC001516	42	24
SQSTM	BC001874	42	25
KCC2D	BC032784	42	26
HPGDS	BC020734	42	28
RGAP1	BC032754	42	29
TFDP1	BC011685	42	30
ZRAB2	BC039814	42	31
PAX3	BC063547	42	32
CREB1	BC010636	42	33
GLYC	BC007979	42	34
CDKN3	BC064965	42	35
SH3L2	BC052987	42	36
SP2	BC033814	42	37
IMA1	BC002374	42	38
DMRT1	BC040847	42	39
IMP4	BC010042	42	40
DCC1	BC001531	42	41
CLK1	BC031549	42	42
NTAQ1	BC008781	42	43
H2A2A	BC096705	42	44

IMA7	BC020520	42	45
NMD3	BC013317	42	46
POGZ	BC057773	42	47
FIBP	BC014388	42	48
NFE2	BC005044	42	49
CELF5	BC028101	42	50
STMN2	BC006302	42	51
FAM9B	BC120955	42	52
NXF3	BC031616	42	53
PP2BA	BC025714	42	54
KAT1	BC021262	42	55
NSD3	BC012059	42	56
LANC1	BC028685	42	57
NAB1	BC035724	42	58
IL37	BC020637	42	59
ICLN	BC119634	42	60
ADAD1	BC040229	42	61
PPM1G	BC000057	42	62
LUC7L	BC065198	42	63
Z385B	BC038422	43	1
G3BP1	BC006997	43	3
TAP26	BC020647	43	4
WAPL	BC017393	43	5
RGS14	BC014094	43	6
SYK	BC004132	43	7
HEXI2	BC025970	43	8
PCBP2	BC001155	43	9
PEX14	BC006327	43	10
HEXDC	BC035561	43	11
RRP15	BC020641	43	12
NOSTN	BC014189	43	13
HJURP	BC001940	43	14
BCL6B	BC059404	43	15
HESX1	BC069515	43	16
MP2K3	BC032478	43	17
AIPL1	BC012055	43	18
PSDE	BC009524	43	19
ZNF69	BC051309	43	20
TFDP2	BC021113	43	21
TRI27	BC013580	43	22
ZN432	BC002858	43	23
MEIS2	BC001516	43	24
SQSTM	BC001874	43	25
KCC2D	BC032784	43	26
HPGDS	BC020734	43	28
RGAP1	BC032754	43	29

TFDP1	BC011685	43	30
ZRAB2	BC039814	43	31
PAX3	BC063547	43	32
CREB1	BC010636	43	33
GLYC	BC007979	43	34
CDKN3	BC064965	43	35
SH3L2	BC052987	43	36
SP2	BC033814	43	37
IMA1	BC002374	43	38
DMRT1	BC040847	43	39
IMP4	BC010042	43	40
DCC1	BC001531	43	41
CLK1	BC031549	43	42
NTAQ1	BC008781	43	43
H2A2A	BC096705	43	44
IMA7	BC020520	43	45
NMD3	BC013317	43	46
POGZ	BC057773	43	47
FIBP	BC014388	43	48
NFE2	BC005044	43	49
CELF5	BC028101	43	50
STMN2	BC006302	43	51
FAM9B	BC120955	43	52
NXF3	BC031616	43	53
PP2BA	BC025714	43	54
KAT1	BC021262	43	55
NSD3	BC012059	43	56
LANC1	BC028685	43	57
NAB1	BC035724	43	58
IL37	BC020637	43	59
ICLN	BC119634	43	60
ADAD1	BC040229	43	61
PPM1G	BC000057	43	62
LUC7L	BC065198	43	63
Z385B	BC038422	44	1
G3BP1	BC006997	44	3
TAP26	BC020647	44	4
WAPL	BC017393	44	5
RGS14	BC014094	44	6
SYK	BC004132	44	7
HEXI2	BC025970	44	8
PCBP2	BC001155	44	9
PEX14	BC006327	44	10
HEXDC	BC035561	44	11
RRP15	BC020641	44	12
NOSTN	BC014189	44	13

HJURP	BC001940	44	14
BCL6B	BC059404	44	15
HESX1	BC069515	44	16
MP2K3	BC032478	44	17
AIPL1	BC012055	44	18
PSDE	BC009524	44	19
ZNF69	BC051309	44	20
TFDP2	BC021113	44	21
TRI27	BC013580	44	22
ZN432	BC002858	44	23
MEIS2	BC001516	44	24
SQSTM	BC001874	44	25
KCC2D	BC032784	44	26
HPGDS	BC020734	44	28
RGAP1	BC032754	44	29
TFDP1	BC011685	44	30
ZRAB2	BC039814	44	31
PAX3	BC063547	44	32
CREB1	BC010636	44	33
GLYC	BC007979	44	34
CDKN3	BC064965	44	35
SH3L2	BC052987	44	36
SP2	BC033814	44	37
IMA1	BC002374	44	38
DMRT1	BC040847	44	39
IMP4	BC010042	44	40
DCC1	BC001531	44	41
CLK1	BC031549	44	42
NTAQ1	BC008781	44	43
H2A2A	BC096705	44	44
IMA7	BC020520	44	45
NMD3	BC013317	44	46
POGZ	BC057773	44	47
FIBP	BC014388	44	48
NFE2	BC005044	44	49
CELF5	BC028101	44	50
STMN2	BC006302	44	51
FAM9B	BC120955	44	52
NXF3	BC031616	44	53
PP2BA	BC025714	44	54
KAT1	BC021262	44	55
NSD3	BC012059	44	56
LANC1	BC028685	44	57
NAB1	BC035724	44	58
IL37	BC020637	44	59
ICLN	BC119634	44	60

ADAD1	BC040229	44	61
PPM1G	BC000057	44	62
LUC7L	BC065198	44	63
CD2B2	BC000495	45	1
HNRPF	BC004254	45	2
DGCR8	BC009323	45	3
RPP40	BC017871	45	4
DAZP1	BC012062	45	5
TOB2	BC038957	45	6
SGT1	BC000721	45	7
T2EB	BC030572	45	8
MAGC2	BC013318	45	9
RNF25	BC015612	45	11
RBBP6	BC029352	45	12
NOC4L	BC001191	45	13
GTPBA	BC021573	45	14
CPNE1	BC001142	45	15
SNAT	BC069434	45	16
RNZ1	BC014624	45	17
SAV1	BC020537	45	18
TCL1A	BC003574	45	19
SNR27	BC017890	45	20
TRAF4	BC001769	45	21
EYA2	BC008803	45	22
ZCHC7	BC036940	45	23
CENPL	BC033154	45	24
SAMN1	BC029112	45	25
PACN1	BC040228	45	26
RL35	BC000348	45	27
NT5C	BC022334	45	28
ZN490	BC038586	45	29
TOX	BC016665	45	30
NUP53	BC047029	45	31
SCC4	BC063863	45	32
ZN439	BC032857	45	33
ABTB1	BC011858	45	34
RS7	BC002866	45	35
BAIP2	BC014020	45	37
ANR54	BC066909	45	39
CPIN1	BC071740	45	40
ST2B1	BC034694	45	41
RHOA	BC005976	45	43
H2A2A	BC096739	45	44
SCMH1	BC021252	45	45
ECM29	BC021127	45	46
VPS4A	BC047932	45	47

Z385C	BC080613	45	48
ZXDC	BC002940	45	49
MEF2C	BC026341	45	50
CTDS2	BC065920	45	51
SSX3	BC103862	45	52
VP33B	BC016445	45	53
NEK3	BC019916	45	54
NOL7	BC023517	45	55
PSME2	BC072025	45	56
ANLN	BC034692	45	57
TRXR1	BC018122	45	58
RP25L	BC032136	45	59
PSA5	BC102018	45	60
ZN363	BC047393	45	61
SAE2	BC003153	45	62
CASP1	BC062327	45	63
IPMK	BC065709	45	64
CD2B2	BC000495	46	1
HNRPF	BC004254	46	2
DGCR8	BC009323	46	3
RPP40	BC017871	46	4
DAZP1	BC012062	46	5
TOB2	BC038957	46	6
SGT1	BC000721	46	7
T2EB	BC030572	46	8
MAGC2	BC013318	46	9
RNF25	BC015612	46	11
RBBP6	BC029352	46	12
NOC4L	BC001191	46	13
GTPBA	BC021573	46	14
CPNE1	BC001142	46	15
SNAT	BC069434	46	16
RNZ1	BC014624	46	17
SAV1	BC020537	46	18
TCL1A	BC003574	46	19
SNR27	BC017890	46	20
TRAF4	BC001769	46	21
EYA2	BC008803	46	22
ZCHC7	BC036940	46	23
CENPL	BC033154	46	24
SAMN1	BC029112	46	25
PACN1	BC040228	46	26
RL35	BC000348	46	27
NT5C	BC022334	46	28
ZN490	BC038586	46	29
TOX	BC016665	46	30

NUP53	BC047029	46	31
SCC4	BC063863	46	32
ZN439	BC032857	46	33
ABTB1	BC011858	46	34
RS7	BC002866	46	35
BAIP2	BC014020	46	37
ANR54	BC066909	46	39
CPIN1	BC071740	46	40
ST2B1	BC034694	46	41
RHOA	BC005976	46	43
H2A2A	BC096739	46	44
SCMH1	BC021252	46	45
ECM29	BC021127	46	46
VPS4A	BC047932	46	47
Z385C	BC080613	46	48
ZXDC	BC002940	46	49
MEF2C	BC026341	46	50
CTDS2	BC065920	46	51
SSX3	BC103862	46	52
VP33B	BC016445	46	53
NEK3	BC019916	46	54
NOL7	BC023517	46	55
PSME2	BC072025	46	56
ANLN	BC034692	46	57
TRXR1	BC018122	46	58
RP25L	BC032136	46	59
PSA5	BC102018	46	60
ZN363	BC047393	46	61
SAE2	BC003153	46	62
CASP1	BC062327	46	63
IPMK	BC065709	46	64
CD2B2	BC000495	47	1
HNRPF	BC004254	47	2
DGCR8	BC009323	47	3
RPP40	BC017871	47	4
DAZP1	BC012062	47	5
TOB2	BC038957	47	6
SGT1	BC000721	47	7
T2EB	BC030572	47	8
MAGC2	BC013318	47	9
RNF25	BC015612	47	11
RBBP6	BC029352	47	12
NOC4L	BC001191	47	13
GTPBA	BC021573	47	14
CPNE1	BC001142	47	15
SNAT	BC069434	47	16

RNZ1	BC014624	47	17
SAV1	BC020537	47	18
TCL1A	BC003574	47	19
SNR27	BC017890	47	20
TRAF4	BC001769	47	21
EYA2	BC008803	47	22
ZCHC7	BC036940	47	23
CENPL	BC033154	47	24
SAMN1	BC029112	47	25
PACN1	BC040228	47	26
RL35	BC000348	47	27
NT5C	BC022334	47	28
ZN490	BC038586	47	29
TOX	BC016665	47	30
NUP53	BC047029	47	31
SCC4	BC063863	47	32
ZN439	BC032857	47	33
ABTB1	BC011858	47	34
RS7	BC002866	47	35
BAIP2	BC014020	47	37
ANR54	BC066909	47	39
CPIN1	BC071740	47	40
ST2B1	BC034694	47	41
RHOA	BC005976	47	43
H2A2A	BC096739	47	44
SCMH1	BC021252	47	45
ECM29	BC021127	47	46
VPS4A	BC047932	47	47
Z385C	BC080613	47	48
ZXDC	BC002940	47	49
MEF2C	BC026341	47	50
CTDS2	BC065920	47	51
SSX3	BC103862	47	52
VP33B	BC016445	47	53
NEK3	BC019916	47	54
NOL7	BC023517	47	55
PSME2	BC072025	47	56
ANLN	BC034692	47	57
TRXR1	BC018122	47	58
RP25L	BC032136	47	59
PSA5	BC102018	47	60
ZN363	BC047393	47	61
SAE2	BC003153	47	62
CASP1	BC062327	47	63
IPMK	BC065709	47	64
CD2B2	BC000495	48	1

HNRPF	BC004254	48	2
DGCR8	BC009323	48	3
RPP40	BC017871	48	4
DAZP1	BC012062	48	5
TOB2	BC038957	48	6
SGT1	BC000721	48	7
T2EB	BC030572	48	8
MAGC2	BC013318	48	9
RNF25	BC015612	48	11
RBBP6	BC029352	48	12
NOC4L	BC001191	48	13
GTPBA	BC021573	48	14
CPNE1	BC001142	48	15
SNAT	BC069434	48	16
RNZ1	BC014624	48	17
SAV1	BC020537	48	18
TCL1A	BC003574	48	19
SNR27	BC017890	48	20
TRAF4	BC001769	48	21
EYA2	BC008803	48	22
ZCHC7	BC036940	48	23
CENPL	BC033154	48	24
SAMN1	BC029112	48	25
PACN1	BC040228	48	26
RL35	BC000348	48	27
NT5C	BC022334	48	28
ZN490	BC038586	48	29
TOX	BC016665	48	30
NUP53	BC047029	48	31
SCC4	BC063863	48	32
ZN439	BC032857	48	33
ABTB1	BC011858	48	34
RS7	BC002866	48	35
BAIP2	BC014020	48	37
ANR54	BC066909	48	39
CPIN1	BC071740	48	40
ST2B1	BC034694	48	41
RHOA	BC005976	48	43
H2A2A	BC096739	48	44
SCMH1	BC021252	48	45
ECM29	BC021127	48	46
VPS4A	BC047932	48	47
Z385C	BC080613	48	48
ZXDC	BC002940	48	49
MEF2C	BC026341	48	50
CTDS2	BC065920	48	51

SSX3	BC103862	48	52
VP33B	BC016445	48	53
NEK3	BC019916	48	54
NOL7	BC023517	48	55
PSME2	BC072025	48	56
ANLN	BC034692	48	57
TRXR1	BC018122	48	58
RP25L	BC032136	48	59
PSA5	BC102018	48	60
ZN363	BC047393	48	61
SAE2	BC003153	48	62
CASP1	BC062327	48	63
IPMK	BC065709	48	64
SPB9	BC002538	49	1
IKZF5	BC022564	49	2
ZMYM3	BC013009	49	3
IFIX	BC020822	49	4
ZN488	BC026895	49	5
CRYAB	BC007008	49	6
ZNF70	BC040161	49	7
OMP	BC069365	49	8
RFC5	BC001866	49	9
RSBN1	BC026155	49	10
IRF6	BC014852	49	11
ZN695	BC055096	49	12
POLH	BC015742	49	13
PRCC	BC004913	49	14
SWP70	BC000616	49	15
H11	BC069492	49	16
ELF3	BC003569	49	17
TDG	BC037557	49	18
ZN343	BC002897	49	19
MPH6	BC031017	49	20
TEX19	BC016939	49	21
REQU	BC014889	49	22
DIDO1	BC000770	49	23
CCNE1	BC035498	49	24
DCAF7	BC001264	49	25
ATX10	BC007508	49	26
FABP6	BC022489	49	27
H2BFS	BC126369	49	28
ARHG8	BC010285	49	29
WDFY3	BC015214	49	30
PPHLN	BC039832	49	31
KCTD1	BC063652	49	32
KIN17	BC017309	49	33

HXA3	BC015180	49	34
HSPB8	BC002673	49	35
H2B1H	BC096116	49	36
PDE1B	BC032226	49	37
ZN502	BC028377	49	38
SASH3	BC051881	49	39
RFC4	BC017452	49	41
ZN223	BC022466	49	42
CLIC1	BC064527	49	43
H2A1	BC104199	49	44
ZN567	BC033849	49	45
SF3A3	BC011523	49	46
CCNJL	BC035871	49	47
SNIP1	BC027040	49	49
FZR	BC013413	49	50
LSM7	BC018621	49	51
AHRR	BC106706	49	52
MBD4	BC011752	49	53
DHX30	BC014237	49	54
OLIG3	BC051352	49	55
ZN276	BC032781	49	57
FOXN3	BC007506	49	58
SUV92	BC029360	49	59
RRMJ2	BC114514	49	60
ZN37A	BC015858	49	61
RUFY2	BC041092	49	62
CDK6	BC052264	49	63
PBX2	BC082261	49	64
SPB9	BC002538	50	1
IKZF5	BC022564	50	2
ZMYM3	BC013009	50	3
IFIX	BC020822	50	4
ZN488	BC026895	50	5
CRYAB	BC007008	50	6
ZNF70	BC040161	50	7
OMP	BC069365	50	8
RFC5	BC001866	50	9
RSBN1	BC026155	50	10
IRF6	BC014852	50	11
ZN695	BC055096	50	12
POLH	BC015742	50	13
PRCC	BC004913	50	14
SWP70	BC000616	50	15
H11	BC069492	50	16
ELF3	BC003569	50	17
TDG	BC037557	50	18

ZN343	BC002897	50	19
MPH6	BC031017	50	20
TEX19	BC016939	50	21
REQU	BC014889	50	22
DIDO1	BC000770	50	23
CCNE1	BC035498	50	24
DCAF7	BC001264	50	25
ATX10	BC007508	50	26
FABP6	BC022489	50	27
H2BFS	BC126369	50	28
ARHG8	BC010285	50	29
WDFY3	BC015214	50	30
PPHLN	BC039832	50	31
KCTD1	BC063652	50	32
KIN17	BC017309	50	33
HXA3	BC015180	50	34
HSPB8	BC002673	50	35
H2B1H	BC096116	50	36
PDE1B	BC032226	50	37
ZN502	BC028377	50	38
SASH3	BC051881	50	39
RFC4	BC017452	50	41
ZN223	BC022466	50	42
CLIC1	BC064527	50	43
H2A1	BC104199	50	44
ZN567	BC033849	50	45
SF3A3	BC011523	50	46
CCNJL	BC035871	50	47
SNIP1	BC027040	50	49
FZR	BC013413	50	50
LSM7	BC018621	50	51
AHRR	BC106706	50	52
MBD4	BC011752	50	53
DHX30	BC014237	50	54
OLIG3	BC051352	50	55
ZN276	BC032781	50	57
FOXN3	BC007506	50	58
SUV92	BC029360	50	59
RRMJ2	BC114514	50	60
ZN37A	BC015858	50	61
RUFY2	BC041092	50	62
CDK6	BC052264	50	63
PBX2	BC082261	50	64
SPB9	BC002538	51	1
IKZF5	BC022564	51	2
ZMYM3	BC013009	51	3

IFIX	BC020822	51	4
ZN488	BC026895	51	5
CRYAB	BC007008	51	6
ZNF70	BC040161	51	7
OMP	BC069365	51	8
RFC5	BC001866	51	9
RSBN1	BC026155	51	10
IRF6	BC014852	51	11
ZN695	BC055096	51	12
POLH	BC015742	51	13
PRCC	BC004913	51	14
SWP70	BC000616	51	15
H11	BC069492	51	16
ELF3	BC003569	51	17
TDG	BC037557	51	18
ZN343	BC002897	51	19
MPH6	BC031017	51	20
TEX19	BC016939	51	21
REQU	BC014889	51	22
DIDO1	BC000770	51	23
CCNE1	BC035498	51	24
DCAF7	BC001264	51	25
ATX10	BC007508	51	26
FABP6	BC022489	51	27
H2BFS	BC126369	51	28
ARHG8	BC010285	51	29
WDFY3	BC015214	51	30
PPHLN	BC039832	51	31
KCTD1	BC063652	51	32
KIN17	BC017309	51	33
HXA3	BC015180	51	34
HSPB8	BC002673	51	35
H2B1H	BC096116	51	36
PDE1B	BC032226	51	37
ZN502	BC028377	51	38
SASH3	BC051881	51	39
RFC4	BC017452	51	41
ZN223	BC022466	51	42
CLIC1	BC064527	51	43
H2A1	BC104199	51	44
ZN567	BC033849	51	45
SF3A3	BC011523	51	46
CCNJL	BC035871	51	47
SNIP1	BC027040	51	49
FZR	BC013413	51	50
LSM7	BC018621	51	51

AHRR	BC106706	51	52
MBD4	BC011752	51	53
DHX30	BC014237	51	54
OLIG3	BC051352	51	55
ZN276	BC032781	51	57
FOXN3	BC007506	51	58
SUV92	BC029360	51	59
RRMJ2	BC114514	51	60
ZN37A	BC015858	51	61
RUFY2	BC041092	51	62
CDK6	BC052264	51	63
PBX2	BC082261	51	64
SPB9	BC002538	52	1
IKZF5	BC022564	52	2
ZMYM3	BC013009	52	3
IFIX	BC020822	52	4
ZN488	BC026895	52	5
CRYAB	BC007008	52	6
ZNF70	BC040161	52	7
OMP	BC069365	52	8
RFC5	BC001866	52	9
RSBN1	BC026155	52	10
IRF6	BC014852	52	11
ZN695	BC055096	52	12
POLH	BC015742	52	13
PRCC	BC004913	52	14
SWP70	BC000616	52	15
H11	BC069492	52	16
ELF3	BC003569	52	17
TDG	BC037557	52	18
ZN343	BC002897	52	19
MPH6	BC031017	52	20
TEX19	BC016939	52	21
REQU	BC014889	52	22
DIDO1	BC000770	52	23
CCNE1	BC035498	52	24
DCAF7	BC001264	52	25
ATX10	BC007508	52	26
FABP6	BC022489	52	27
H2BFS	BC126369	52	28
ARHG8	BC010285	52	29
WDFY3	BC015214	52	30
PPHLN	BC039832	52	31
KCTD1	BC063652	52	32
KIN17	BC017309	52	33
HXA3	BC015180	52	34

HSPB8	BC002673	52	35
H2B1H	BC096116	52	36
PDE1B	BC032226	52	37
ZN502	BC028377	52	38
SASH3	BC051881	52	39
RFC4	BC017452	52	41
ZN223	BC022466	52	42
CLIC1	BC064527	52	43
H2A1	BC104199	52	44
ZN567	BC033849	52	45
SF3A3	BC011523	52	46
CCNJL	BC035871	52	47
SNIP1	BC027040	52	49
FZR	BC013413	52	50
LSM7	BC018621	52	51
AHRR	BC106706	52	52
MBD4	BC011752	52	53
DHX30	BC014237	52	54
OLIG3	BC051352	52	55
ZN276	BC032781	52	57
FOXN3	BC007506	52	58
SUV92	BC029360	52	59
RRMJ2	BC114514	52	60
ZN37A	BC015858	52	61
RUFY2	BC041092	52	62
CDK6	BC052264	52	63
PBX2	BC082261	52	64
CENPN	BC008972	53	1
PPM1A	BC026691	53	2
IRF5	BC004139	53	3
NUMB	BC020788	53	4
MK12	BC015741	53	5
MBIP1	BC005197	53	6
TF3C2	BC000212	53	7
MEMO1	BC018733	53	8
TRIB2	BC002637	53	9
ZN232	BC016039	53	10
DNJC7	BC011837	53	11
H2AZ	BC018002	53	12
ST17B	BC016040	53	13
ZN131	BC035875	53	15
MAD1	BC069377	53	16
HXC10	BC001293	53	17
KC1E	BC006490	53	18
MYCBP	BC008686	53	19
VHL	BC058831	53	20

ZN136	BC006421	53	21
CREST	BC034494	53	22
HNRPR	BC001449	53	23
VRK3	BC023556	53	24
ZBT37	BC003116	53	25
FYV1	BC032389	53	26
ATF6A	BC018064	53	27
NEDD8	BC104201	53	28
ZN100	BC035579	53	29
FBW1B	BC026213	53	30
PSB5	BC057840	53	31
AN32B	BC013003	53	32
KLF12	BC019680	53	33
MPIP3	BC019089	53	34
CK054	BC007110	53	35
T53G3	BC098165	53	36
RAGP1	BC014044	53	37
AP5Z1	BC037399	53	38
SIAH1	BC042550	53	39
PHF6	BC005994	53	40
MAGC2	BC005891	53	41
FOXA1	BC033890	53	42
MYOME	BC005949	53	43
SYNCI	BC119700	53	44
ZN548	BC030788	53	45
KKCC1	BC031647	53	46
PAR6B	BC060847	53	47
WWTR1	BC014052	53	49
EGR2	BC035625	53	50
RPB11	BC024165	53	51
FOXR1	BC125040	53	52
EIF3L	BC029265	53	53
IKZF3	BC032707	53	54
RPR1A	BC000225	53	55
WDR44	BC028697	53	56
VPS72	BC003151	53	57
GLYM	BC032584	53	58
RNH2C	BC023588	53	59
GFRP	BC112262	53	60
N42L2	BC010643	53	61
NSUN2	BC001041	53	62
DYXC1	BC062564	53	63
UBP49	BC014176	53	64
CENPN	BC008972	54	1
PPM1A	BC026691	54	2
IRF5	BC004139	54	3

NUMB	BC020788	54	4
MK12	BC015741	54	5
MBIP1	BC005197	54	6
TF3C2	BC000212	54	7
MEMO1	BC018733	54	8
TRIB2	BC002637	54	9
ZN232	BC016039	54	10
DNJC7	BC011837	54	11
H2AZ	BC018002	54	12
ST17B	BC016040	54	13
ZN131	BC035875	54	15
MAD1	BC069377	54	16
HXC10	BC001293	54	17
KC1E	BC006490	54	18
MYCBP	BC008686	54	19
VHL	BC058831	54	20
ZN136	BC006421	54	21
CREST	BC034494	54	22
HNRPR	BC001449	54	23
VRK3	BC023556	54	24
ZBT37	BC003116	54	25
FYV1	BC032389	54	26
ATF6A	BC018064	54	27
NEDD8	BC104201	54	28
ZN100	BC035579	54	29
FBW1B	BC026213	54	30
PSB5	BC057840	54	31
AN32B	BC013003	54	32
KLF12	BC019680	54	33
MPIP3	BC019089	54	34
CK054	BC007110	54	35
T53G3	BC098165	54	36
RAGP1	BC014044	54	37
AP5Z1	BC037399	54	38
SIAH1	BC042550	54	39
PHF6	BC005994	54	40
MAGC2	BC005891	54	41
FOXA1	BC033890	54	42
MYOME	BC005949	54	43
SYNCI	BC119700	54	44
ZN548	BC030788	54	45
KKCC1	BC031647	54	46
PAR6B	BC060847	54	47
WWTR1	BC014052	54	49
EGR2	BC035625	54	50
RPB11	BC024165	54	51

FOXR1	BC125040	54	52
EIF3L	BC029265	54	53
IKZF3	BC032707	54	54
RPR1A	BC000225	54	55
WDR44	BC028697	54	56
VPS72	BC003151	54	57
GLYM	BC032584	54	58
RNH2C	BC023588	54	59
GFRP	BC112262	54	60
N42L2	BC010643	54	61
NSUN2	BC001041	54	62
DYXC1	BC062564	54	63
UBP49	BC014176	54	64
CENPN	BC008972	55	1
PPM1A	BC026691	55	2
IRF5	BC004139	55	3
NUMB	BC020788	55	4
MK12	BC015741	55	5
MBIP1	BC005197	55	6
TF3C2	BC000212	55	7
MEMO1	BC018733	55	8
TRIB2	BC002637	55	9
ZN232	BC016039	55	10
DNJC7	BC011837	55	11
H2AZ	BC018002	55	12
ST17B	BC016040	55	13
ZN131	BC035875	55	15
MAD1	BC069377	55	16
HXC10	BC001293	55	17
KC1E	BC006490	55	18
MYCBP	BC008686	55	19
VHL	BC058831	55	20
ZN136	BC006421	55	21
CREST	BC034494	55	22
HNRPR	BC001449	55	23
VRK3	BC023556	55	24
ZBT37	BC003116	55	25
FYV1	BC032389	55	26
ATF6A	BC018064	55	27
NEDD8	BC104201	55	28
ZN100	BC035579	55	29
FBW1B	BC026213	55	30
PSB5	BC057840	55	31
AN32B	BC013003	55	32
KLF12	BC019680	55	33
MPIP3	BC019089	55	34

CK054	BC007110	55	35
T53G3	BC098165	55	36
RAGP1	BC014044	55	37
AP5Z1	BC037399	55	38
SIAH1	BC042550	55	39
PHF6	BC005994	55	40
MAGC2	BC005891	55	41
FOXA1	BC033890	55	42
MYOME	BC005949	55	43
SYNCI	BC119700	55	44
ZN548	BC030788	55	45
KKCC1	BC031647	55	46
PAR6B	BC060847	55	47
WWTR1	BC014052	55	49
EGR2	BC035625	55	50
RPB11	BC024165	55	51
FOXR1	BC125040	55	52
EIF3L	BC029265	55	53
IKZF3	BC032707	55	54
RPR1A	BC000225	55	55
WDR44	BC028697	55	56
VPS72	BC003151	55	57
GLYM	BC032584	55	58
RNH2C	BC023588	55	59
GFRP	BC112262	55	60
N42L2	BC010643	55	61
NSUN2	BC001041	55	62
DYXC1	BC062564	55	63
UBP49	BC014176	55	64
CENPN	BC008972	56	1
PPM1A	BC026691	56	2
IRF5	BC004139	56	3
NUMB	BC020788	56	4
MK12	BC015741	56	5
MBIP1	BC005197	56	6
TF3C2	BC000212	56	7
MEMO1	BC018733	56	8
TRIB2	BC002637	56	9
ZN232	BC016039	56	10
DNJC7	BC011837	56	11
H2AZ	BC018002	56	12
ST17B	BC016040	56	13
ZN131	BC035875	56	15
MAD1	BC069377	56	16
HXC10	BC001293	56	17
KC1E	BC006490	56	18

MYCBP	BC008686	56	19
VHL	BC058831	56	20
ZN136	BC006421	56	21
CREST	BC034494	56	22
HNRPR	BC001449	56	23
VRK3	BC023556	56	24
ZBT37	BC003116	56	25
FYV1	BC032389	56	26
ATF6A	BC018064	56	27
NEDD8	BC104201	56	28
ZN100	BC035579	56	29
FBW1B	BC026213	56	30
PSB5	BC057840	56	31
AN32B	BC013003	56	32
KLF12	BC019680	56	33
MPIP3	BC019089	56	34
CK054	BC007110	56	35
T53G3	BC098165	56	36
RAGP1	BC014044	56	37
AP5Z1	BC037399	56	38
SIAH1	BC042550	56	39
PHF6	BC005994	56	40
MAGC2	BC005891	56	41
FOXA1	BC033890	56	42
MYOME	BC005949	56	43
SYNCI	BC119700	56	44
ZN548	BC030788	56	45
KKCC1	BC031647	56	46
PAR6B	BC060847	56	47
WWTR1	BC014052	56	49
EGR2	BC035625	56	50
RPB11	BC024165	56	51
FOXR1	BC125040	56	52
EIF3L	BC029265	56	53
IKZF3	BC032707	56	54
RPR1A	BC000225	56	55
WDR44	BC028697	56	56
VPS72	BC003151	56	57
GLYM	BC032584	56	58
RNH2C	BC023588	56	59
GFRP	BC112262	56	60
N42L2	BC010643	56	61
NSUN2	BC001041	56	62
DYXC1	BC062564	56	63
UBP49	BC014176	56	64
PTN2	BC016727	57	1

UBXN6	BC008288	57	2
NR0B1	BC011564	57	3
TPIS	BC017917	57	4
PXK	BC014479	57	5
DLX6	BC069363	57	8
LMCD1	BC000646	57	9
LYAR	BC015796	57	10
UBQL4	BC006410	57	11
DPOE4	BC031331	57	12
PSD12	BC019062	57	13
DMRTD	BC029202	57	14
CPSF6	BC000714	57	15
DRG1	BC020803	57	16
ZSC16	BC004255	57	17
ACD11	BC019607	57	18
RPAB2	BC003582	57	19
FOPNL	BC022321	57	20
ZN440	BC035760	57	21
ZNF92	BC036439	57	22
ATMIN	BC002701	57	23
CSTF1	BC001011	57	24
RTCA	BC012604	57	25
PPARG	BC006811	57	26
RGS13	BC016667	57	27
C42S2	BC096703	57	28
CRY1	BC030519	57	29
ZN257	BC036446	57	30
IN80B	BC050666	57	31
ARGL1	BC071587	57	32
NSF1C	BC002801	57	33
GATA2	BC015613	57	34
AICDA	BC006296	57	35
H2B1D	BC096122	57	36
TNIP1	BC014008	57	37
PIAS4	BC010047	57	38
KLF17	BC049844	57	39
MAT2B	BC005218	57	40
PHAX	BC021161	57	41
ZN639	BC020500	57	42
BTBDA	BC005071	57	43
CDX1	BC096252	57	44
MYNN	BC033620	57	45
UAP1	BC009377	57	46
ETV6	BC043399	57	47
ZN607	BC014850	57	49
CCNA1	BC036346	57	50

TX1B3	BC023980	57	51
DTD1	BC100923	57	52
ZN417	BC025783	57	53
RS27	BC002658	57	54
ERCC1	BC052813	57	55
CREB3	BC009402	57	57
FEZF2	BC022464	57	58
TNNI2	BC032148	57	59
COMD6	BC117391	57	60
SIR1	BC012499	57	61
VDR	BC060832	57	62
DCP2	BC064593	57	63
PTN2	BC016727	58	1
UBXN6	BC008288	58	2
NR0B1	BC011564	58	3
TPIS	BC017917	58	4
PXK	BC014479	58	5
DLX6	BC069363	58	8
LMCD1	BC000646	58	9
LYAR	BC015796	58	10
UBQL4	BC006410	58	11
DPOE4	BC031331	58	12
PSD12	BC019062	58	13
DMRTD	BC029202	58	14
CPSF6	BC000714	58	15
DRG1	BC020803	58	16
ZSC16	BC004255	58	17
ACD11	BC019607	58	18
RPAB2	BC003582	58	19
FOPNL	BC022321	58	20
ZN440	BC035760	58	21
ZNF92	BC036439	58	22
ATMIN	BC002701	58	23
CSTF1	BC001011	58	24
RTCA	BC012604	58	25
PPARG	BC006811	58	26
RGS13	BC016667	58	27
C42S2	BC096703	58	28
CRY1	BC030519	58	29
ZN257	BC036446	58	30
IN80B	BC050666	58	31
ARGL1	BC071587	58	32
NSF1C	BC002801	58	33
GATA2	BC015613	58	34
AICDA	BC006296	58	35
H2B1D	BC096122	58	36

TNIP1	BC014008	58	37
PIAS4	BC010047	58	38
KLF17	BC049844	58	39
MAT2B	BC005218	58	40
PHAX	BC021161	58	41
ZN639	BC020500	58	42
BTBDA	BC005071	58	43
CDX1	BC096252	58	44
MYNN	BC033620	58	45
UAP1	BC009377	58	46
ETV6	BC043399	58	47
ZN607	BC014850	58	49
CCNA1	BC036346	58	50
TX1B3	BC023980	58	51
DTD1	BC100923	58	52
ZN417	BC025783	58	53
RS27	BC002658	58	54
ERCC1	BC052813	58	55
CREB3	BC009402	58	57
FEZF2	BC022464	58	58
TNNI2	BC032148	58	59
COMD6	BC117391	58	60
SIR1	BC012499	58	61
VDR	BC060832	58	62
DCP2	BC064593	58	63
PTN2	BC016727	59	1
UBXN6	BC008288	59	2
NR0B1	BC011564	59	3
TPIS	BC017917	59	4
PXK	BC014479	59	5
DLX6	BC069363	59	8
LMCD1	BC000646	59	9
LYAR	BC015796	59	10
UBQL4	BC006410	59	11
DPOE4	BC031331	59	12
PSD12	BC019062	59	13
DMRTD	BC029202	59	14
CPSF6	BC000714	59	15
DRG1	BC020803	59	16
ZSC16	BC004255	59	17
ACD11	BC019607	59	18
RPAB2	BC003582	59	19
FOPNL	BC022321	59	20
ZN440	BC035760	59	21
ZNF92	BC036439	59	22
ATMIN	BC002701	59	23

CSTF1	BC001011	59	24
RTCA	BC012604	59	25
PPARG	BC006811	59	26
RGS13	BC016667	59	27
C42S2	BC096703	59	28
CRY1	BC030519	59	29
ZN257	BC036446	59	30
IN80B	BC050666	59	31
ARGL1	BC071587	59	32
NSF1C	BC002801	59	33
GATA2	BC015613	59	34
AICDA	BC006296	59	35
H2B1D	BC096122	59	36
TNIP1	BC014008	59	37
PIAS4	BC010047	59	38
KLF17	BC049844	59	39
MAT2B	BC005218	59	40
PHAX	BC021161	59	41
ZN639	BC020500	59	42
BTBDA	BC005071	59	43
CDX1	BC096252	59	44
MYNN	BC033620	59	45
UAP1	BC009377	59	46
ETV6	BC043399	59	47
ZN607	BC014850	59	49
CCNA1	BC036346	59	50
TX1B3	BC023980	59	51
DTD1	BC100923	59	52
ZN417	BC025783	59	53
RS27	BC002658	59	54
ERCC1	BC052813	59	55
CREB3	BC009402	59	57
FEZF2	BC022464	59	58
TNNI2	BC032148	59	59
COMD6	BC117391	59	60
SIR1	BC012499	59	61
VDR	BC060832	59	62
DCP2	BC064593	59	63
PTN2	BC016727	60	1
UBXN6	BC008288	60	2
NR0B1	BC011564	60	3
TPIS	BC017917	60	4
PXK	BC014479	60	5
DLX6	BC069363	60	8
LMCD1	BC000646	60	9
LYAR	BC015796	60	10

UBQL4	BC006410	60	11
DPOE4	BC031331	60	12
PSD12	BC019062	60	13
DMRTD	BC029202	60	14
CPSF6	BC000714	60	15
DRG1	BC020803	60	16
ZSC16	BC004255	60	17
ACD11	BC019607	60	18
RPAB2	BC003582	60	19
FOPNL	BC022321	60	20
ZN440	BC035760	60	21
ZNF92	BC036439	60	22
ATMIN	BC002701	60	23
CSTF1	BC001011	60	24
RTCA	BC012604	60	25
PPARG	BC006811	60	26
RGS13	BC016667	60	27
C42S2	BC096703	60	28
CRY1	BC030519	60	29
ZN257	BC036446	60	30
IN80B	BC050666	60	31
ARGL1	BC071587	60	32
NSF1C	BC002801	60	33
GATA2	BC015613	60	34
AICDA	BC006296	60	35
H2B1D	BC096122	60	36
TNIP1	BC014008	60	37
PIAS4	BC010047	60	38
KLF17	BC049844	60	39
MAT2B	BC005218	60	40
PHAX	BC021161	60	41
ZN639	BC020500	60	42
BTBDA	BC005071	60	43
CDX1	BC096252	60	44
MYNN	BC033620	60	45
UAP1	BC009377	60	46
ETV6	BC043399	60	47
ZN607	BC014850	60	49
CCNA1	BC036346	60	50
TX1B3	BC023980	60	51
DTD1	BC100923	60	52
ZN417	BC025783	60	53
RS27	BC002658	60	54
ERCC1	BC052813	60	55
CREB3	BC009402	60	57
FEZF2	BC022464	60	58

TNNI2	BC032148	60	59
COMD6	BC117391	60	60
SIR1	BC012499	60	61
VDR	BC060832	60	62
DCP2	BC064593	60	63
ATAD2	BC019909	61	1
ZN561	BC032668	61	2
CLK2	BC014067	61	3
CDCA4	BC025263	61	4
CSK22	BC008812	61	6
NRBP	BC001221	61	7
USF1	BC035505	61	8
DUS12	BC006286	61	9
SUV91	BC006238	61	10
TRIA1	BC002638	61	11
AFTIN	BC022247	61	12
SSX1	BC128611	61	13
ACTB	BC016045	61	14
APLF	BC041144	61	15
CASPE	BC069541	61	16
SURF6	BC014878	61	17
UBP21	BC003130	61	18
BIRC5	BC008718	61	19
8ODP	BC014618	61	20
RBMS1	BC012992	61	22
MED1	BC060758	61	23
EHD4	BC006287	61	24
SRSF6	BC006832	61	25
RBM42	BC002868	61	26
HEN1	BC013789	61	27
SPNXD	BC103961	61	28
NF2L2	BC011558	61	29
HKDC1	BC021278	61	30
ABT1	BC066313	61	31
RPP30	BC006991	61	32
P53	BC003596	61	33
TF2AY	BC025991	61	34
RSLAA	BC058077	61	35
H2B1O	BC106720	61	36
MTF2	BC010013	61	37
PRAME	BC014074	61	38
TAF5L	BC041094	61	39
ZBT38	BC008901	61	40
PRS8	BC001932	61	41
BRSK1	BC016681	61	42
LEG3	BC053667	61	43

CENPX	BC009571	61	46
HSFY1	BC055414	61	47
NACA2	BC062710	61	48
RBMX	BC006550	61	49
XIAP	BC032729	61	50
RAB8B	BC020654	61	51
ZN586	BC124558	61	52
YAF2	BC037777	61	53
GMEB1	BC001473	61	54
HAT1	BC063003	61	55
PCBP2	BC071942	61	56
NR2E1	BC028031	61	57
TOX2	BC007636	61	58
MND1	BC032142	61	59
H1FNT	BC118635	61	60
ORC2	BC014834	61	61
REC8	BC004159	61	62
CARD8	BC056891	61	63
NUP50	BC070133	61	64
ATAD2	BC019909	62	1
ZN561	BC032668	62	2
CLK2	BC014067	62	3
CDCA4	BC025263	62	4
CSK22	BC008812	62	6
NRBP	BC001221	62	7
USF1	BC035505	62	8
DUS12	BC006286	62	9
SUV91	BC006238	62	10
TRIA1	BC002638	62	11
AFTIN	BC022247	62	12
SSX1	BC128611	62	13
ACTB	BC016045	62	14
APLF	BC041144	62	15
CASPE	BC069541	62	16
SURF6	BC014878	62	17
UBP21	BC003130	62	18
BIRC5	BC008718	62	19
8ODP	BC014618	62	20
RBMS1	BC012992	62	22
MED1	BC060758	62	23
EHD4	BC006287	62	24
SRSF6	BC006832	62	25
RBM42	BC002868	62	26
HEN1	BC013789	62	27
SPNXD	BC103961	62	28
NF2L2	BC011558	62	29

HKDC1	BC021278	62	30
ABT1	BC066313	62	31
RPP30	BC006991	62	32
P53	BC003596	62	33
TF2AY	BC025991	62	34
RSLAA	BC058077	62	35
H2B1O	BC106720	62	36
MTF2	BC010013	62	37
PRAME	BC014074	62	38
TAF5L	BC041094	62	39
ZBT38	BC008901	62	40
PRS8	BC001932	62	41
BRSK1	BC016681	62	42
LEG3	BC053667	62	43
CENPX	BC009571	62	46
HSFY1	BC055414	62	47
NACA2	BC062710	62	48
RBMX	BC006550	62	49
XIAP	BC032729	62	50
RAB8B	BC020654	62	51
ZN586	BC124558	62	52
YAF2	BC037777	62	53
GMEB1	BC001473	62	54
HAT1	BC063003	62	55
PCBP2	BC071942	62	56
NR2E1	BC028031	62	57
TOX2	BC007636	62	58
MND1	BC032142	62	59
H1FNT	BC118635	62	60
ORC2	BC014834	62	61
REC8	BC004159	62	62
CARD8	BC056891	62	63
NUP50	BC070133	62	64
ATAD2	BC019909	63	1
ZN561	BC032668	63	2
CLK2	BC014067	63	3
CDCA4	BC025263	63	4
CSK22	BC008812	63	6
NRBP	BC001221	63	7
USF1	BC035505	63	8
DUS12	BC006286	63	9
SUV91	BC006238	63	10
TRIA1	BC002638	63	11
AFTIN	BC022247	63	12
SSX1	BC128611	63	13
ACTB	BC016045	63	14

APLF	BC041144	63	15
CASPE	BC069541	63	16
SURF6	BC014878	63	17
UBP21	BC003130	63	18
BIRC5	BC008718	63	19
8ODP	BC014618	63	20
RBMS1	BC012992	63	22
MED1	BC060758	63	23
EHD4	BC006287	63	24
SRSF6	BC006832	63	25
RBM42	BC002868	63	26
HEN1	BC013789	63	27
SPNXD	BC103961	63	28
NF2L2	BC011558	63	29
HKDC1	BC021278	63	30
ABT1	BC066313	63	31
RPP30	BC006991	63	32
P53	BC003596	63	33
TF2AY	BC025991	63	34
RSLAA	BC058077	63	35
H2B1O	BC106720	63	36
MTF2	BC010013	63	37
PRAME	BC014074	63	38
TAF5L	BC041094	63	39
ZBT38	BC008901	63	40
PRS8	BC001932	63	41
BRSK1	BC016681	63	42
LEG3	BC053667	63	43
CENPX	BC009571	63	46
HSFY1	BC055414	63	47
NACA2	BC062710	63	48
RBMX	BC006550	63	49
XIAP	BC032729	63	50
RAB8B	BC020654	63	51
ZN586	BC124558	63	52
YAF2	BC037777	63	53
GMEB1	BC001473	63	54
HAT1	BC063003	63	55
PCBP2	BC071942	63	56
NR2E1	BC028031	63	57
TOX2	BC007636	63	58
MND1	BC032142	63	59
H1FNT	BC118635	63	60
ORC2	BC014834	63	61
REC8	BC004159	63	62
CARD8	BC056891	63	63

NUP50	BC070133	63	64
ATAD2	BC019909	64	1
ZN561	BC032668	64	2
CLK2	BC014067	64	3
CDCA4	BC025263	64	4
CSK22	BC008812	64	6
NRBP	BC001221	64	7
USF1	BC035505	64	8
DUS12	BC006286	64	9
SUV91	BC006238	64	10
TRIA1	BC002638	64	11
AFTIN	BC022247	64	12
SSX1	BC128611	64	13
ACTB	BC016045	64	14
APLF	BC041144	64	15
CASPE	BC069541	64	16
SURF6	BC014878	64	17
UBP21	BC003130	64	18
BIRC5	BC008718	64	19
8ODP	BC014618	64	20
RBMS1	BC012992	64	22
MED1	BC060758	64	23
EHD4	BC006287	64	24
SRSF6	BC006832	64	25
RBM42	BC002868	64	26
HEN1	BC013789	64	27
SPNXD	BC103961	64	28
NF2L2	BC011558	64	29
HKDC1	BC021278	64	30
ABT1	BC066313	64	31
RPP30	BC006991	64	32
P53	BC003596	64	33
TF2AY	BC025991	64	34
RSLAA	BC058077	64	35
H2B1O	BC106720	64	36
MTF2	BC010013	64	37
PRAME	BC014074	64	38
TAF5L	BC041094	64	39
ZBT38	BC008901	64	40
PRS8	BC001932	64	41
BRSK1	BC016681	64	42
LEG3	BC053667	64	43
CENPX	BC009571	64	46
HSFY1	BC055414	64	47
NACA2	BC062710	64	48
RBMX	BC006550	64	49

XIAP	BC032729	64	50
RAB8B	BC020654	64	51
ZN586	BC124558	64	52
YAF2	BC037777	64	53
GMEB1	BC001473	64	54
HAT1	BC063003	64	55
PCBP2	BC071942	64	56
NR2E1	BC028031	64	57
TOX2	BC007636	64	58
MND1	BC032142	64	59
H1FNT	BC118635	64	60
ORC2	BC014834	64	61
REC8	BC004159	64	62
CARD8	BC056891	64	63
NUP50	BC070133	64	64

Supplementary Table 2: Primers used in site directed mutagenesis procedure.

Gene	Amino acid mutation	Sense primer (5'-3')	Antisense primer (5'-3')
Hck	K290E	CTGGCTTCATCGTCTCCACTG CCACCTTGGT	ACCAAGGTGGCAGTGGAGA CGATGAAGCCAG
Ror2	K507E	CTTTGTCCTTCAGCGTCTCGAT GGCCACAGCCTGG	CCAGGCTGTGGCCATCGAGA CGCTGAAGGACAAAG
Btk	K430E	GCCTTCTTTGATCATCTCGAT GGCCACGTCGTACT	AGTACGACGTGGCCATCGAG ATGATCAAAGAAGGC
Frk	K262R	GAACCTGGTTTTAATGTTCTCA CTGCTACTGGAGTGG	CCACTCCAGTAGCAGTGAGA ACATTAAAACCAGGTT
Ksyk	K396R	GTATTTTCACGCCACGGTTC TCACCACTTTTTTCATTT GGTAGTA	TACTACCAAATGAAAAAAG TTGTGAGAACCGTGGCT GTGAAAATAC
Fgfr1	K512R	GACCAAGGCTGTGAGGATG TTGAAGTCGGACG	CGTCCGACTTCAACATCCT CACAGCCACTTTGGTC
Ptk6	K219M	CGAGAAATCACCATAATGGC CACCTGGACCCG	CGGGTCCAGGTGGCCATTA TGGTGATTTCTCG

Supplementary Table 3: Primers used for assembly PCR

Primer Name	Primer Sequence (5'-3')
5'GW223-c-Myc	CTCGAGAATTCGCCACCATGGAACAAAAATTGATAAGTGAGGAAGATTTGTACAAAAAAGTTGGCATG
3'GW223-His	GTAGCAGCCTGAGTCGTTATTAGTGATGGTGATGGTGATGGCCAACCTTGTACAAGAAAGTTGG
5'UTR	GATCTTAAGGCTAGAGTACTAATACGACTCACTATAGGGAATACAAGCTACTTGTTCTTTTTGCACTCGAGAATTCGCCACCATG
3'UTR	CAAAAAACCCCTCAAGACCCGTTTAGAGGCCCCAAGGGGTTATGCTAGTTTTTTTTTTTTTTTTTTTTTTTTTTTTTTGTAGCAGCCTGAGTCG
5'Final	GATCTTAAGGCTAGAGTAC
3'Final	CAAAAAACCCCTCAAGAC
